# Green Nanofabrication Opportunities in the Semiconductor Industry: A Life Cycle Perspective

**DOI:** 10.3390/nano11051085

**Published:** 2021-04-22

**Authors:** Eleanor Mullen, Michael A. Morris

**Affiliations:** CRANN and AMBER Research Centres, School of Chemistry, Trinity College Dublin, D02 W085 Dublin, Ireland

**Keywords:** area selective deposition (ASD), directed self-assembly (DSA), block copolymer (BCP), polymer brushes, lithography, green chemistry, semiconductor industry, environmental sustainability, life cycle assessment (LCA) and ex ante analysis, bottom-up lithography, top-down lithography, nanofabrication

## Abstract

The turn of the 21st century heralded in the semiconductor age alongside the Anthropocene epoch, characterised by the ever-increasing human impact on the environment. The ecological consequences of semiconductor chip manufacturing are the most predominant within the electronics industry. This is due to current reliance upon large amounts of solvents, acids and gases that have numerous toxicological impacts. Management and assessment of hazardous chemicals is complicated by trade secrets and continual rapid change in the electronic manufacturing process. Of the many subprocesses involved in chip manufacturing, lithographic processes are of particular concern. Current developments in bottom-up lithography, such as directed self-assembly (DSA) of block copolymers (BCPs), are being considered as a next-generation technology for semiconductor chip production. These nanofabrication techniques present a novel opportunity for improving the sustainability of lithography by reducing the number of processing steps, energy and chemical waste products involved. At present, to the extent of our knowledge, there is no published life cycle assessment (LCA) evaluating the environmental impact of new bottom-up lithography versus conventional lithographic techniques. Quantification of this impact is central to verifying whether these new nanofabrication routes can replace conventional deposition techniques in industry as a more environmentally friendly option.

## 1. Introduction 

The fabrication of micro/nano structures and devices in the semiconductor industry requires the deposition of thin films, followed by lithographic patterning techniques and etching to remove specific regions of the material [1,2]. Lithographic techniques have been central to the exponential miniaturization of electronics over the past 50 years. However, the techno-economic model that underpins Moore’s law is beginning to fail due to the increased fabrication costs associated with transistors reaching atomic scales [3]. To address new miniaturisation challenges, developments in lithography should have sufficiently defined geometries, enable production of miniaturised features, be time efficient and have low production costs [4,5,6,7]. Bottom-up lithography techniques may offer an alternative fabrication route to conventional lithography that circumvents these challenges [7,8,9,10].

Two bottom-up techniques are discussed in this review: area selective deposition (ASD) of polymer brushes and DSA of BCPs. Briefly, ASD allows for alignment of materials at a pre-patterned surface in replacement of a lithographic mask [7]. This is achieved by allowing a film to be deposited in one region of a surface while simultaneously avoiding deposition in other regions to achieve ASD [11]. In the case of DSA, inorganic material infiltrates self-assembled molecular blocks forming patterns such as nanodot arrays or nanowires post-polymer removal [12]. It is the chemical incompatibility of the BCP blocks that enables self-assembly into a variety of morphologies [13,14,15]. The molecular mechanisms of ASD and DSA technologies must be better understood before these techniques can be integrated into semiconductor manufacturing processes [11,16,17,18].

Both ASD and DSA are bottom-up fabrication approaches whereby atoms and/or molecules are assembled into desired structures [7,13,19,20,21]. The advantage of this approach lies in its atomic precision, facilitating the fabrication of structures with atomic or molecular size resolutions [19,22,23]. This methodology differs from the current top-down fabrication approach that has been used in the semiconductor industry for decades, and has seen an almost exponential growth in some cases [19,24]. Top-down approaches refer to the performance of some physical processes such as cutting or slicing to transform a bulk material into nanoparticles or materials with controlled shapes and sizes [22]. Nonetheless, top-down approaches such as extreme ultraviolet lithography (EUVL) and nanoimprint lithography (NIL) have limitations. Key challenges for next generation EUVL and NIL technologies include finding suitable resist materials, source power, overlay and defect repair process time [16,22,25]. The implementation of bottom-up BCP and brush lithographic techniques in semiconductor production could circumvent the associated complexity and expense of the photolithographic and multiple patterning lithography etch and deposition steps [7]. 

**Figure 1:**
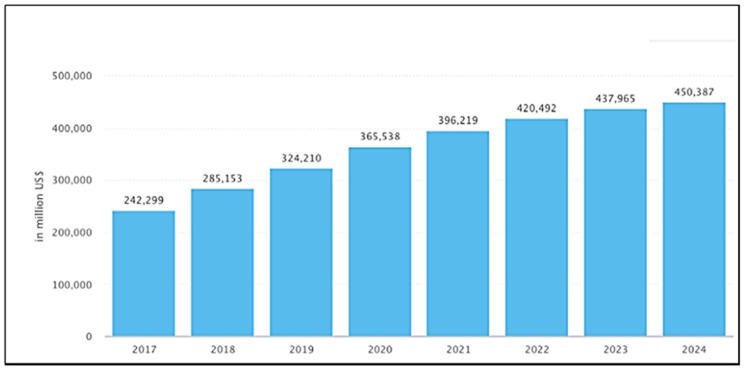
Consumer electronics growth in USD up to 2019 and the projected growth after 2019 [33]. Original source ([33]) followed by the IEEE copyright line © 2020 IEEE.

Thus, there is an economic incentive driving the development of BCP and brush lithography techniques but is there an environmental incentive? The development of industries such as the electronics industry has led to perturbations in environmental system (ES) functioning; if left unchecked, such issues may pose a serious threat to the future sustainability of human life on earth [26]. The demand for modern integrated circuits (ICs) has led to sales in the semiconductor industry increasing by almost seven times in the past three decades from $73,000 in 1987 to $481,090 in 2018 [27,28]. This growth of consumer electronics is illustrated in Figure 1 below. The ever expanding role semiconductors play in our society has led to an increase in the use of potentially hazardous chemicals such as the metals, photoactive chemicals, toxic gases, organic solvents and acids required for manufacturing [29]. The volume of this market is such that any small improvement in the electronics life cycle leads to a dramatic increase in environmental sustainability [30,31,32]. 

Lithography requires the greatest number of chemicals amongst the semiconductor fabrication processes [34,35]. The main set of chemicals used during photolithography are photoresists. They consist of a polymer, solvent, sensitizer, and additional additives. BCP and brush lithographic techniques do not require a sensitizer or these additional additives and therefore may reduce chemical wastes. Production costs will also be reduced if the need for a photolithographic mask is circumvented [34]. It has been suggested that BCP and brush lithography provide rapid processing, scalable synthesis, and reduced production costs when compared to current lithographic techniques [4,36]. Currently less energy is required to power machinery for ASD and DSA deposition technologies. For example, ASD has been shown to work at relatively low temperature conditions that can be achieved using simple machinery [36,37]. However, this technology is still at a developmental stage and has not yet reached an industrial level [4]. The environmental and economic benefits of this technology have yet to be quantified. How much will expenditure be reduced by if photolithographic masks are no longer used? How would avoiding the use of chemicals such as perfluorocarbons (PFCs) in photolithographic processes affect the amount of hazardous waste products in factories? How will this impact wildlife and local communities? How much power would be saved if top-down lithography was replaced by bottom-up nanofabrication techniques?

We suggest a life cycle analysis (LCA) framework for quantifying the impact of ASD and DSA in industry. A life cycle refers to the time span to produce a finished product from the extraction of the raw materials, its manufacture, use, and its disposal requirements [38]. LCA at the early stage of technological development can be used to provide decision making support for the selection of environmentally preferable alternatives [39]. For the first time an LCA approach is suggested as a means to evaluate the environmental and economic impact of bottom up lithographic techniques before industrial scale implementation. Additionally, LCA can be used to further improve and integrate sustainability practices into a technology before its use on vast scales, by which time major changes require too much effort [40,41]. Therefore, if LCA techniques are employed during the research and development phases of these new routes to nanofabrication, environmental burdens and investment errors can be avoided. 

Several challenges must be overcome in order to perform an LCA that compares BCP and brush lithography with currently used top-down lithographic techniques. Firstly, the complexity of the semiconductor devices means that the fabrication process is intricate, and the mass of the chemicals and water used in the production far outweighs the mass of the end-product device. This makes LCA of electronics more challenging than that of plastics, for example [42]. Secondly, BCP and brush nanofabrication techniques are at the research stage of development, therefore LCA is more challenging to implement. LCA was originally developed for the study of systems where there was enough information about material and energy inputs and output and the cause and effect relationships through the supply chain. Applying LCA to emerging technologies is therefore challenging [43]. Drawing comparisons between brush and BCP nanofabrication routes and current lithographic techniques is especially challenging due to the prevalence of commercial secrecy in the semiconductor industry [29]. Nevertheless, applying LCA methodologies at this early stage of development is crucial to ensuring informed decision making that evaluates environmental risks, and ensures that new regulations and natural resource constraints do not lead to delays and unforeseeable increases in costs [26,44,45]. Thirdly, methodologies such as LCA exist to characterise the environmental impacts of new technologies but there is no internationally recognised LCA methodology to monitor the environmental consequences of the microelectronics industry [46]. Finally, there is no available literature focusing specifically on the LCA of BCP and brush lithography techniques in the semiconductor industry. However, publications on LCA of the semiconductor industry do exist and have become more numerous since the 2000s [47,48,49,50]. 

This study is divided into three main sections. The first section discusses how BCP and polymer brush lithography compare to existing lithographic techniques in terms of environmental sustainability. The following section discusses material challenges in environmental, safety, health, and sustainability (ESH/S) in the semiconductor industry, and emerging BCP and brush-based nanofabrication techniques that can be integrated into the semiconductor industry to address some of these challenges. Finally, the last section develops a framework for evaluating the LCA of BCPs and brush lithography strategies in the semiconductor industry. Using an LCA framework to evaluate environmental sustainability is central to enabling researchers to direct future green chemistry transitions in semiconductor production. This is of particular importance at the research and design phase where such transitions can mitigate severe ecological impacts, reduce waste generation and energy costs, and alleviate the hazards associated with the usage of novel materials such as nanoparticles. The framework is limited by the lack of accurate information and literature concerning the life cycle of lithographic processes. It is found that future studies should examine how barriers to LCA such as commercial secrecy in the semiconductor industry could be overcome.

## 2. Bottom-Up versus Top-Down Lithography 

The investigation of alternative lithographic processes is central to the cost effective progression of the industry [51,52,53]. It is also an opportune moment to consider environmental sustainability and future environmental regulatory requirements that may restrict industrial development and increase costs [33,40,49,54,55]. DSA is considered, in the international technology roadmap for semiconductors, as a patterning strategy for next generation lithography [56]. Both ASD and DSA facilitate the pattering of a material with a reduced number of processing steps [4,7,36]. Section 2.1 outlines the stages required for BCP and brush lithography, but how do they compare to top-down lithographic approaches? Can they provide cost effective environmental solutions to improve manufacturing processes? In Section 2.2 we compare popular top-down nanomanufacturing routes with those that use BCP and brush lithographic technologies. 

### 2.1. Block Copolymer and Polymer Brush Lithography

BCP and brush nanofabrication techniques begin with polymer selection, followed by polymer deposition onto the substrate, polymer patterning, metal incorporation and ending with an oxidative removal of polymer and metal reduction. 

#### 2.1.1. Polymer Selection

The structure of BCP films depends on the molecular architecture, weight and composition of the BCP chosen [57]. The simplest BCPs are linear di-block copolymers for example polystyrene-block-poly(methyl methacrylate) (PS-b-PMMA).They consist of two polymeric blocks, joined by a covalent bond [1,58]. BCPs form a highly ordered pattern of nanodomains under suitable conditions [59]. The size and structural arrangement of the domains is determined by the volume and molecular weight fraction of the blocks selected [58]. The resulting self-assembled nanodomains can consist of morphologies ranging from cylinders to gyroids to spheres, depending on the predetermined self-assembly conditions [60]. In BCP lithography, one block can be selectively removed to leave an on-substrate etch masks; alternatively block selective insertion of metals from the vapour or solution phase can be used to create masks where both polymers can be removed simultaneously [61,62]. This ability to control specific properties such as morphology and feature size is the main advantage of BCP lithography as an aid for optical patterning of device elements for future IC developments [1,63,64].

Polymer brushes can be defined as long chain polymer molecules that are attached to an interface or surface by some means [65]. They can be used for ASD to facilitate selective patterning of large substrate areas [11,17]. For example, in poly(2-vinylpyridine) (P2VP) or poly(4-vinylpyridine) (P4VP) brushes, it is the presence of an unshared electron pair on the nitrogen atom of the pyridine ring that facilitates coordination bonding with various metal species [66]. Conversely, Polymer brushes such as polystyrene (PS) can be used for surface deactivation to block metals adhering to the substrate [17,21,67,68]. 

Presently BCPs and polymer brushes are selected based on their ability to produce high quality patterned substrates for specific applications such as in the semiconductor industry. To enable the transition of BCPs and polymer brushes from “lab to fab” research efforts have focused on finding suitable BCPs with controllable ordination of the phase separated domains and long-range alignment and polymer brushes with high uniformity (pinhole free) complete coverage over large areas and tuneable thickness [18,69]. However, it is worth considering the importance of green polymer chemistry and the potential of this new technology to be environmentally friendly if toxic moieties are substituted for biocompatible materials. The natural world offers us a nearly infinite selection of available polymers or precursor monomers to choose from for polymer deposition. It is probable that bio-based polymers may become a go-to in the near future due to their biodegradability, as industries become more aware of their waste management [70,71]. Incorporating environmental sustainability considerations into research efforts directed at the transition of new technologies from “lab to fab” will help satisfy future environmental restrictions and economic success in markets [44]. 

Perhaps for example, more environmentally friendly green brush alternatives could be considered for example, PEG or polypyrrole thin films [72]. Many possibilities exist for green BCPs such as Amino-acid derived copolymers. An example being the work of Miyazaki et al. to generate polyethylene glycol-poly(amino acid) (PEG-PAA) BCPs [73]. Rabotyagova et al. also discuss the possible biosynthesis opportunities for protein based BCP synthesis [74]. Furthermore, it is possible to produce nanopatterned protein-polysaccharide thin films as reported by Banta and co-workers [75]. However, further work is required before they can be utilised to the same extent as conventional BCP systems for DSA. 

Fatty acids do not have polar functional groups throughout their structure, but they possess end carboxylic acid groups and, as such, can be adapted for blocking surfaces when patterning metals. Stearic acid, oleic acids and various others have already been used as surface coatings mainly for nanoparticle stabilisation, however due to their inherent hydrophobic nature, we propose their application to ASD for surface blocking [76,77]. Stearyl and lauryl acrylate were utilised by Wang et al. to design a bio-based block copolymer with styrene moieties as a more sustainable thermoplastic [78]. Once again further research is required before these polymers can be utilised to the same extent as conventional polymer brush deposition techniques for ASD. 

Other biodegradable polymer possibilities include chitosan [79,80], soybean plastics [81], zein [82], alginates [83], PEG [84], and other wood and plant derived polymers [85,86], among various others [87]. For example, polypyrolle or poly(vinyl pyrrolidone) can be used in BCP development due to their biocompatibility and biodegradability properties [88,89,90]. With careful selection of polymer chemistry, it may be possible to design a suitable biodegradable system for BCP and polymer brush lithographic techniques. However further research is necessary to determine if this is possible. Many advances have been made in developing the use of Biodegradable block copolymers in the medical industry [91]. Perhaps expansion of this research into the semiconductor industry may be beneficial to environmental sustainability. 

Nevertheless, bio-based polymers also have environmental implications such as emissions during extraction and production as well as manufacturing costs. For example, the production of polylactic acid (PLA) generates CO_2_ emissions and chemical wastes during its extraction [92]. It is possible however using green chemistry principles to reduce environmental impacts during the production and extraction of bio-based polymers. Morão and co-workers discussed the LCA assessment of PLA, whereby increasing sugarcane yield, reducing chemical waste and employing renewable energy alternatives during PLA production can reduce the CO_2_ footprint almost two-fold [93]. 

It is also important to consider the possibility of chemically recycling polymer wastes. For example the recycling process of chemical depolymerisation of PLA to produce lactic acid and get back PLA [94]. The production of polymers should aim to reduce the amount of organic solvents being used or improve the quality of their recyclability. It is the use of solvents in polymer production that produces the largest quantities of auxiliary waste [95].

#### 2.1.2. Polymer Deposition onto Substrates 

After the selection of a suitable polymer for polymer brush ASD or BCP DSA, a method for depositing the polymeric material onto the substrate needs to be selected. Common deposition techniques include dip-coating or spin-coating a flat wafer substrate with a precursor polymer solution. These liquid phase deposition techniques do not need specialist equipment with high power requirements [37,96,97,98]. 

Other techniques, however, do not employ liquid solvents as a deposition medium. One example of a solvent-free method is chemical vapour deposition (CVD) which uses a chemical reaction and/or dissociation of gaseous reactants to deposit a solid onto a heated surface [99,100,101,102]. This technique can be used to deposit polymer films onto a substrate. For example, highly cross-linked homopolymer coatings, ultrathin films, and designs of unique copolymers are achievable using CVD, whereby precursor monomers react in vapor form [99,103]. From a polymer physics perspective, the process can be easier to control and may circumvent concerns about surface de-wetting effects [104]. CVD avoids solvent use, and also has the economic benefit of being typically a fast, efficient, one step process, with film growth rates of >100 nm sec^−1^ [21]. However the high temperature and vacuum environment required for precursors to be volatile for CVD is more energy intensive than liquid deposition methods [105]. 

#### 2.1.3. Polymer-Based Pattern Formation 

**Figure 2:**
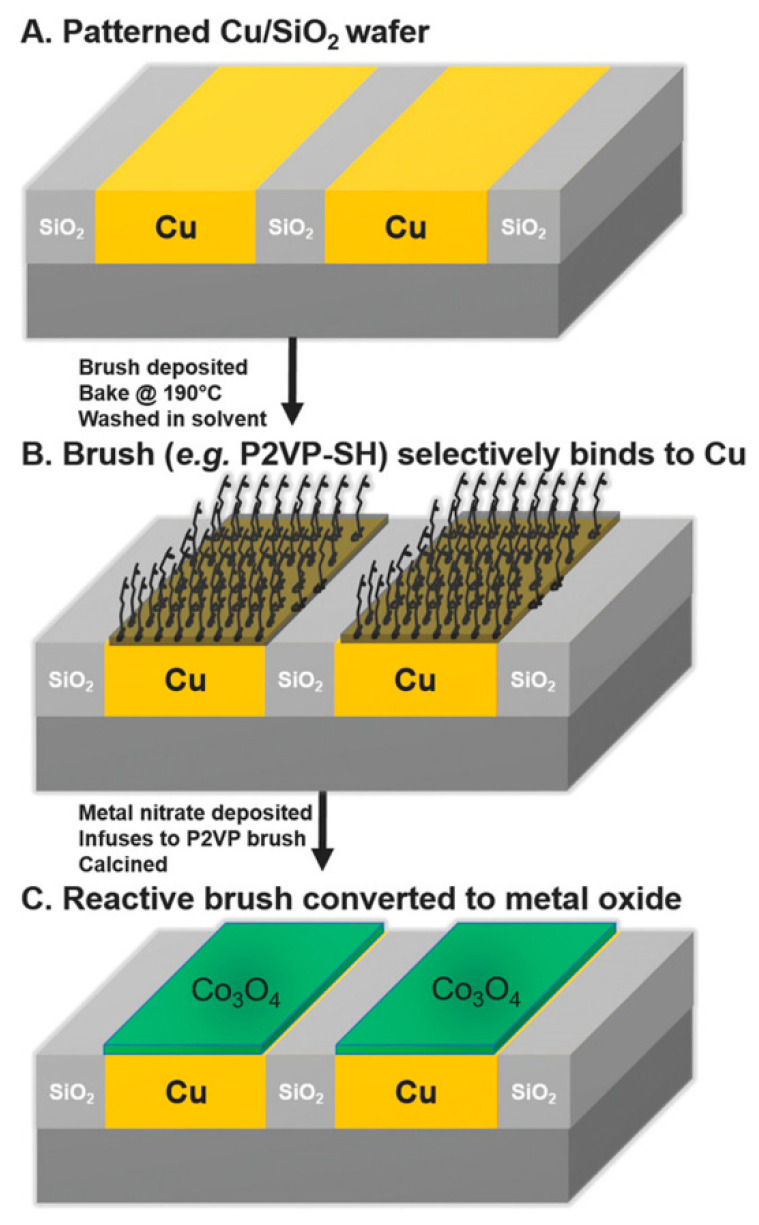
An example of ASD of P2VP-SH brush onto a Cu/SiO_2_ wafer and subsequent metal oxide patterning [7]. Image reproduced with permission from Wiley [7].

ASD as mentioned previously can be used for polymer-based pattern formation to activate or deactivate a surface region. The capability of polymer brushes to poses high grafting densities make them an appealing option for surface activation or deactivation [17]. The quality of polymer brush deposition or attachment to the substate requires carful optimisation to achieve monolayer formation, which determines the regularity of the inorganic film [21]. Selecting the chemistry of the end-functional group of the polymer brushes facilitates selective substrate affinity and thus selective ASD [7,106]. For example, Cummins et al. used hydroxy-terminated brushes that tend to preferably bind to the Cu regions selectively over SiO_2_ substrates. The metal nitrate then infiltrates the polymer brush resulting in selective deposition of dense metal films [18]. Hence, a combination of various end-terminated brushes can be used to pattern substrates and engineer semiconductor surfaces, as outlined in Figure 2. Polymer brushes have versatile application and also enable the tailoring of the substrate surface energies that can influence BCP morphology [15]. 

Pattern formation of BCPs requires DSA of the polymer blocks into ordered nanodomains [107,108]. There are various routes to DSA: solvent vapour annealing (SVA) [109,110], thermal annealing [111], laser-initiated self-assembly, atomic layer deposition (ALD) and various others [4].

SVA improves the kinetics of self-assembly and is often performed at ambient temperatures thus requiring low power source temperature control [14]. The sustainability of this approach is dependent on selecting an environmentally friendly solvent and polymer [112]. Solvents used in DSA include toluene and tetrahydrofuran (THF), but there are also green solvents such as water, ethanol, or ethyl lactate [113,114,115,116]. It is therefore feasible to select an appropriate green solvent such as ethyl lactate, for example, as an alternative to ethyl acetate and acetone [117,118]. There are extensive resources available to support the selection of an appropriate green solvent for a desired application [119,120,121]. Ionic liquids (ILs) can also be environmentally friendly if chosen carefully [122]. Development of an industrial SVA system that is fast and precise, with potential for wafer scale processing, will help facilitate the transition of this technology from the research scale to the industrial [123,124,125]. Annealing chambers with temperature and gas flow systems facilitate thickness and pattern formation control [124,125]. BCP patterns of 5 to 200 nm size range with dot, line hole or lamellar patterns have a predicted use in HMV (High volume manufacturing) in 2022 in device type: 3 nm node logic [16,123,126].

Thermal annealing facilitates the production of microphase structures during DSA and is achieved by raising the temperature just below the order disorder temperature(ODT) [115]. Energy cost depends on the ODT required for the process [115]. Laser-initiated self-assembly is very rapid but high costs can be incurred depending on laser power [127,128]. ALD can also be used. This process consists of sequential alternating pulses of gaseous precursor chemicals that react with the substrate, where the number of sequential cycles determines the film thickness [129]. ALD can be used to precisely modify nanoscale BCP features on a large scale, leading to conformal and well-organised hybrid-pattern nanoscale pattern formation [14,126]. 

Considerable interest lies in the development of alternative processes for patterning and the self-alignment of materials that will cut manufacturing costs [16]. DSA of BCPs can be achieved at low cost using very basic and cheap laboratory apparatus [130]. Short processing periods make for an efficient production rate [7].

#### 2.1.4. Metal Incorporation and Oxidative Removal of the Polymer 

ASD of polymer brushes and DSA of BCPs facilitates the patterned deposition of inorganic materials. Deposition of inorganic materials into polymer brush matrix or self-assembled nanodomains of BCPs can also be achieved using liquid deposition processes such as spin coating [11,18,130,131]. Other methods of metal inclusion are ALD and low temperature vapour phase deposition and sequential infiltration (SIS) [12,62,132]. 

ALD can also be used in ASD for vapour phase deposition of a metal ion into a polymer brush [133]. ASD facilitates selective blocking and activation of substrate areas. When ALD is used to deposit metal ions the thickness can be actively controlled and deposited only in activated areas of the substrate [7]. Integrating polymer brush deposition methodologies with defined metal deposition methods such as ALD and CVD requires further research. 

**Figure 3:**
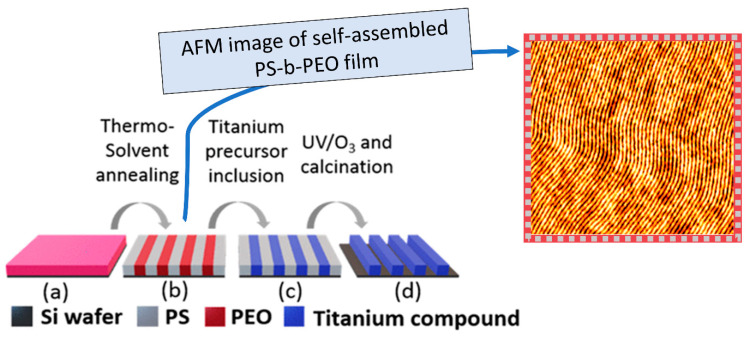
Illustration of the formation of titanium dioxide nanowires. (**a**) Si substrate with an as-cast film of PS-b-PEO solution, (**b**) post thermo-solvent vapour annealing self-assembled PS-b-PEO is achieved. (**c**) film after titanium precursor inclusion. (**d**) post UV ozone and calcination process titanium dioxide nanowires. AFM image (2 × 2 µm^2^) of the self-assembled PS-b-PEO thin film is shown on the furthest right. Reprinted (adapted) with permission from ([37]). Copyright (2018) American Chemical Society.

Alternatively, low vapour phase deposition can be used to deposit inorganic material into BCP’s self-assembled nanodomains, or polymer brush films onto silicon substrates [21,37]. For example, TiO_2_ nanowires can be produced by microphase separation of polystyrene-block-poly(ethylene oxide) (PS-*b*-PEO) to produce self-assembled arrangement of horizontal pillars (see Figure 3 part (b) below) [37]. The AFM image (furthest right of Figure 3) shows a 2 × 2 µm^2^ surface typographical images of the PS-b-PEO thin film acting as a template. For example, low temperature CVD can subsequently be used to selectively deposit titanium tetraisopropoxide (TTIP) into the block copolymer thin film template, as illustrated in Figure 3 part (c) [37,104]. Many metals, such as iron [107], nickel [134], copper [17], zinc [134], silver [135], gold [136], and others can also be deposited *by* metal ion precursor in ethanol solution, or low temperature vapour deposition [14,130]. The low-temperature nature of the vapour phase deposition process may eliminate the energy costs and the expenses associated with the employment of complex machinery and optical systems [137].

Post metal infiltration UV/ozone treatment and calcination can be used to remove the organic components and leave behind an array of TiO_2_ surface nanowires (Figure 3 part d) [37,107]. Processing time is less than two hours, yielding titanium dioxide nanostructures. Oxidative processes are commonly used in both ASD and DSA to remove the polymer templates in order to produce homogeneous metal and metal oxide thin films [7,21,125].

We can therefore conclude that DSA and ASD can create integrated architectures for metal infiltration and are an alternative inexpensive approach to sub-20 nm lithography. There are many benefits to this polymer based lithographic approach such as: no diffraction limit in resolution, directly patterned functional materials, efficiency in 3D patterning, possible improved feature control to sub 7 nm and pitch multiplication, reduction of processing steps and an innovative self-aligned patterning process with the capability to extend to 3D architectures [133,137,138]. These techniques have a predicted low cost of ownership and do not require the use of photo resists and so avoid acquisition costs such as resist spinner hotplates (estimated cost of $44,000), or deep UV photoresist stabilization systems (estimated cost of $57,000), along with their associated installation costs [139]. 

However, there are some limitations; reduced defect density of DSA of BCPs is required, Additionally the range of metals that can be deposited via these lithographic methods requires expansion [138]. For example, deposition methods such as ALD and CVD require reductive process to produce metal materials post polymer removal and have a limited range of metals that can deposited. ALD and CVD also have poor etch contrast of polymer blocks that limits quality of device structures post pattern transfer and requires improvement [7,14,140]. A standardised methodology for polymer based lithographic techniques that has effective defect control, effective pattern transfer and flexible nanopatterning is critical [10,138]. Defect mitigation and repair techniques are under development as they are simple, consisting of basic apparatus for pressure and temperature control [123,124].

### 2.2. Comparison of the Next-Generation Lithographic Techniques

BCP and brush nanofabrication techniques have yet to be adopted into IC manufacturing due to several challenges related to pattern defects and pattern placement [141]. Results are largely based on studies by academic institutions [16]. However, many of these experiments are performed without sufficient control of all parameters. 

ASD and DSA techniques are compared in this subsection to conventional optical lithography, extreme EUVL, and NIL, to illustrate the opportunities that BCP and brush lithography present. Table 1 and Table 2 compare and summarize the characteristics and relative resolution and feature sizes of the nanofabrication techniques. EUVL and NIL are selected because they are aggressive candidates in the race to obtain half pitches less than 20nm [142]. Optical lithography is selected because it is most widely used [143]. There are many patterning techniques under development that can be compared to BCP and brush lithography, but detailing this in full here would be beyond the scope of the review.

#### 2.2.1. Optical/Photo Lithography

**Figure 4:**
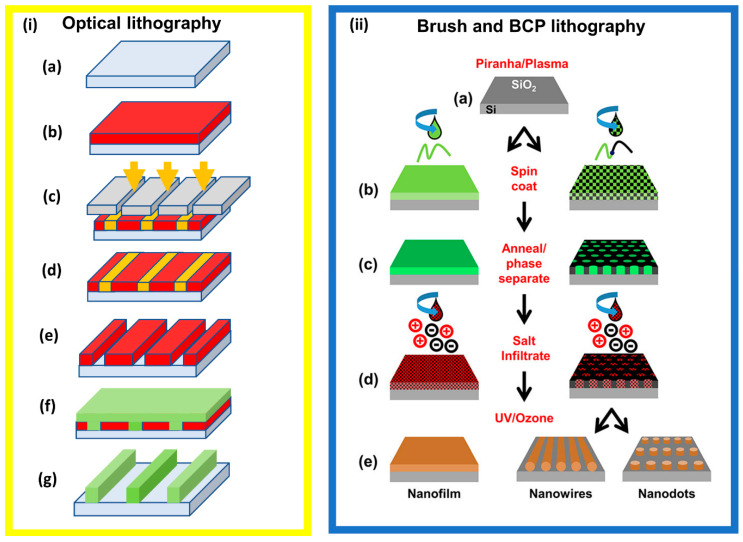
Comparison of (**i**) optical lithography and (**ii**) brush and BCP lithography. The first stage for both these processes is (**a**) substrate cleaning. The basic steps for optical lithography illustrated in Figure 4 part (**i**) and consist of, (**b**) photoresist deposition, (**c**) UV exposure, (**d**) post-baking, (**e**) development, (**f**) metal thin film deposition, and (**g**) polishing and etching [150]. The principal steps for Brush and BCP lithography of polymers are shown in Figure 4 part (**ii**) and comprise of (**b**) polymer spin casting, (**c**) annealing/phase separation, (**d**) salt infiltration, and (**e**) UV/ozone [18]. Reprinted (adapted) with permission from ([18]). Copyright (2010) American Chemical Society.

Optical lithography is a photon-based technique. It is the most widely used lithographic route in the semiconductor industry for the manufacture of nano-electronics [144,145,146]. The process typically involves substrate coating with a photoresist, and placement of a photomask on top, whereby upon exposure to UV or visible light the photomask would change the solubility of photoresist in exposed regions [147]. Chemical development is then used to selectively dissolve these regions of altered solubility, thereby leaving holes in the photoresist behind [5,147]. The result can then either be used to engrave a pattern into exposed regions of the material, for ion implantation, or metal deposition of a new material using select chemical treatments [148]. This stage of wafer processing is energy intensive and requires a greater number of chemicals than other processes [35,149]. Figure 4. Illustrates the differences between optical lithography, and BCP and brush lithography techniques (ASD and DSA).

ASD and DSA can achieve pattern transfer with a reduced number of steps [21,51]. For example, optical lithography requires UV exposure of a photomask to lay down the foundation for pattern formation, whereas DSA only requires phase separation or annealing of the polymer film [51]. This readily demonstrates the advantage of DSA, since once the polymer film has undergone annealing, the pattern is developed and ready for metal inclusion. Contrarily, optical lithography still requires post-baking and chemical development before it is ready for metal inclusion. Therefore, optical lithography is a chemically intensive process [35].

Photolithography uses high volumes of chemicals at each of its processing stages, and is a cause for environmental concern [34]. Photoresists are light sensitive materials that undergo photochemical reactions on exposure to light [57]. Constituents include chemicals such as propylene glycol monomethyl ether acetate (PGMEA) and ether acetate as carrier solvents [149]. PGMEA is one of the most important solvents for photoresist processing in the semiconductor industry. It has relatively low environmental toxicity but all of the reactants required in its manufacture are from non-renewable sources [151]. Carcinogens used in photoresists (PR) products include cyclohexanone, ethylbenzene, pyridine, and 1,4 dioxane [34]. Cyclohexanone has central nervous system inhibitory and aesthetic reactions [152]. Photoresists, after exposure to light, decompose to low–molecular weight compounds, such as phenol, cresol, benzene, toluene, xylene, and other benzene-based aromatic compounds, in addition to the carrier solvents [149]. Benzene is known to have deleterious health and environmental effects [153]. Phenolic compounds are toxic to fish and other aquatic organisms, and are listed by the US environmental protection agency (USEPA) as priory pollutants [152].

Optical lithography is reaching the minimum resolution that can be achieved, as described by the Rayleigh equation [137,144]. Traditionally this issue was resolved by reducing the exposure wavelength, by improving the photoresist material and by further development of optical illumination techniques [144,145,154,155], but photolithography can no longer be further optimised as it has an intrinsic resolution limit [51].

Non-radiative patterning techniques such as ASD and DSA are less mature techniques but potentially circumvent these issues by not having a diffraction limit in resolution, and by patterning directly onto functional materials [137,156]. For example, optimising the sensitivity of photoresists to imaging radiation is no longer a requirement [137]. 

CVD is often used for metal thin film deposition into patterned areas defined by conventional lithography and ASD or DSA deposition. CVD in the semiconductor industry requires regular cleaning of tool chambers. Cleaning is achieved through the use of PFCs which has led to an increase in their consumption since the 1980s [157,158]. These PFCs are classified as organohalogen contaminants and are found in both wildlife and human tissues, including breast milk [159,160]. Under normal operating conditions, as much as 10 to 80 percent of PFCs pass through the manufacturing tool chambers unreacted and are released into the air [157]. BCP and polymer brush techniques present an opportunity to eliminate or reduce their use. Another issue with current CVD methodologies is that high purity gaseous reactants or carrier gases are required for CVD [102]. Nitrogen is a favourable alternative to CVD as securing stable helium supplies become increasingly short in supply [161]. CVD systems involved in DSA currently are modelled on Nitrogen carrier gases [123].

Current costs associated with setting up a start-up nanofabrication/characterisation facility in terms of site acquisition, service contract, and installation costs comes to a total cost of 65.8 million US dollars. This is 42% of the total start-up costs, of which 9.86 million USD is associated with lithography and 18.12 million USD is associated with lithography support [139]. Lithography is perhaps the most critical processing step to improve since it has such high capital equipment cost [52]. The cost of lithographic masks is becoming a dominant cost in lithography, and so adding to the appeal of the ASD and the DSA techniques [52]. 

More sophisticated methodologies to replace optical lithography are being developed. Here ASD and DSA are also compared to these next generation lithography techniques to ascertain their technological competitiveness. The International Roadmap for Devices and Systems (IRDS) Lithography 2020 edition lists the following next generation technology (NGT) for lithography:; extreme ultraviolet (EUV) single patterning, EUV multiple patterning, high-NA EUV (EUV with a 0.55 NA lens), EUV new wavelength, Nanoimprint lithography, and DSA [16]. In our discussion we group all EUV techniques together and suggest an additional NGT called ASD.

#### 2.2.2. Extreme Ultraviolet Lithography

EUVL is an extension of optical lithography that specifically employs light in the EUV region of 13.5 nm [144,162,163]. EUVL is used for its efficient development and manufacturing cycle time, increased number of patterning levels, and its addressing the overall complexity of extending multiple patterning into higher multiples [33]. Recent EUV tools have enabled a possible productive capacity of 125 wafers per hour [164]. EUV technology is still relatively modern and therefore it relies on specific and costly multilayer reflective optics, a high vacuum, and hot 20–50 eV plasma or accelerator technology for the production of EUV radiation [51,154,165]. The power required for the 13.5 nm light source is estimated to be 200 W for 125 wafers of 300 mm size per hour. EUV technology is expensive to install with a tool cost of more than 30 million USD [137,142,144]. Defect mitigation and repair techniques are expensive, costly and time consuming [166]. Making this high power process economical is one of the most challenging obstacles that must be overcome for the use of EUVL in industry [16,154,167]. Furthermore, EUVL typically requires extensive use of chemicals: thermoplastic, molecular glass or fullerene derived resists, and acid generators and acid amplifiers [163,168,169]. The more precise the process the more likely a longer list of chemicals would be required, adding to the complexity of environmental risk assessments. Improved line edge roughness, high etch resistance development cycle time, manufacturing cycle time, increased number of patterning levels, and increased sensitivity are some of the attributes EUVL needs to acquire to realise its full potential as a main candidate for sub-10-nm manufacturing [33,51,138,170].

#### 2.2.3. Nanoimprint Lithography

NIL is a mechanical process for patterning wafers where the mould physically deforms a photoresist layer prior to cross-linking [137,144,171]. Once the resist is cured the mould is removed and the patterned resist is used for nanomanufacturing [171]. This process usually requires toxic and non-degradable photoresists consisting of the polymer matrix, photoactive compounds and cross-linkers [149,172]. Polymer brush and BCP lithographic techniques that circumvent the need for photoresist processing stages can avoid wastes generated by the use of photoresist and photostripper [142,146,171]. 

NIL does however have many merits including the parallel processing of large area substrates which facilitates a change in flash memory from scaling horizontally to vertically [16,173]. Additionally, its processing steps have high throughput, low cost and high resolution, and it allows for patterning features of sub 100 nm -possibly with features as small as 10 nm [51,137,146,174]. Further developments are required though to reduce process steps, improve fabrication quality and mass production capacity, overlay accuracy, reduce defectivity, improve inspection and defect repair techniques [16,33]. Additionally, issues with mould materials need to be resolved. Moulds made of rigid materials enable the support of fine features as small as 5nm, but this leads to high defect rates. Moulds of elastomeric materials can be used to address this challenge but the higher flexibility leads to new defects due to thermal expansion [171,175]. The technique is Limited by size of features that can be patterned on the mould and has high fidelity of pattern transfer [137]. Tool complexity depends on the kind of NIL technique used for example thermal embossing NIL (TE-NIL) uses heat and pressure during pattern transfer, and UV nanoimprint lithography (SFIL) uses capillary forces, pressure, and light exposure [174]. Improved tool performance is required for mass production [173,176].

## 3. Material Challenges in Environmental, Safety, Health, and Sustainability in the Semiconductor Industry

This section opens with a discussion on the ever more pressing environmental issues the semiconductor industry faces. It identifies how the replacement of conventional lithographic techniques with bottom up nanofabrication techniques could help meet environmental regulations governing the electronics life cycle. The section concludes with the answer to the question we posed in the introduction: are there environmental incentives to pursue the alternative lithography strategies?

### 3.1. Pressing Enviornmental Issues the Semicoductor Industry Faces

#### 3.1.1. Reducing Waste

**Figure 5:**
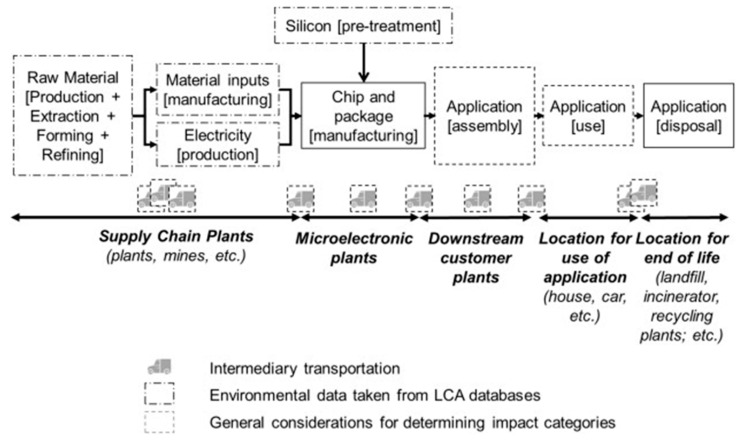
The general phases in a component life cycle [46]. Reprinted from Journal of Cleaner Production, Volume 86, Aurélie Villard, Alan Lelah, Daniel Brissaud, Drawing a chip environmental profile: environmental indicators for the semiconductor industry, Pages 98–109., Copyright (2015), with permission from Elsevier [46].

The lifecycle of electronics is complex with an environmental footprint spread across many different industrial stages as can be seen in Figure 5 below [178]. Extraction and supply of raw materials is the first stage of waste generation [179]. This Figure illustrates how the remainder of the life cycle naturally splits into semiconductor production processes and their applications [46].

The semiconductor production process comprises deposition, resist coating, light exposure, etching and removal of the resist and rinsing which generates a considerable amount of hazardous waste [180]. Advancements towards finer patterning and larger wafer sizes have led to increases in water, chemical and material requirements [35,56,181] The quality of waste management practices in the semiconductor industry varies and commonly contains a range of toxic pollutants such as solvents, arsenic, fine oxide particles, salts, alkalis, inorganic compounds and pure organic compounds [180]. The effect of material wastes such as metals and their accumulation in ecosystems is of critical environmental concern [182]. A study by Suzuki et al. in 2007 found that gallium, arsenic, indium and titanium were found in Taiwanese squirrels living in areas surrounding semiconductor fabrication plants [183]. There is a lack of publications on metal accumulation in the environment from semiconductor fabrication plants.

Recovery and reuse of water, acids and other chemicals would help resolve waste issues [178,184]. However, the purity of recovered waste might be compromised which makes recovery not always profitable [178]. Additionally, equipment might only be used for a few years before being replaced with new tools due to changes in the semiconductor production processes [32,185]. Consumable electronics generally get disposed of within a few years [28].

#### 3.1.2. Reducing Power Consumption

Each process tool in a lab consumes energy either directly during its operation or indirectly through other facilities or sub-systems (e.g., process vacuum systems or compressed dry air) [186,187]. Manufacturing equipment for more complex semiconductors, larger diameter wafers and the requirement for improved cleanrooms have vastly increased the energy consumption [188]. Intermediary transportation of raw materials, wastes and products requires further power consumption [179]. The worldwide energy demand has doubled during the last 30 years as a result of growing population, expanding industry and globalisation [189]. Global climate change concerns are driving efforts to reduce emissions of greenhouse gases in semiconductor manufacturing [190]. Future limits on energy could also potentially limit the industry’s ability to build or expand new factories [189].

#### 3.1.3. Avoiding Threats to Earth Systems

The development of novel technologies requires an understanding of how to design chemical processes that avoid molecular mechanisms which could pose a threat to Earth systems [191]. Earth systems can be threatened by the use of a chemical in industry if these three circumstances occur: (i) the chemical has an unknown distributive impact on a vital system process, (ii) only when this distributive effect becomes a global scale problem is it realised, (iii) the effect is not easily reversed [26]. 

An example of a chemical used in the semiconductor industry that poses a threat to Earth systems are PFCs. Mentioned previously, PFCs are used in photolithographic processes and are organ halogen contaminants [192]. The use of organ halogen compounds (OHCs) in, for example, pesticides, flame retardants and hydraulic fluids before their environmental toxicity was known has effected species in some of the world’s most isolated places [159]. PFCs contribute to global warming and have bio accumulative and toxic properties [40,193]. Thus, the use of PFCs satisfies the above criteria for a chemical to pose a threat to an Earth system. The distributive impact is being realised now that the chemical has become a global scale problem, but the exact impact is known. PFCs are in the food chain: so the effect of this chemical use is not readably reversible and affecting wildlife in some of the world’s most isolated regions. For example, Polar bears (*ursus maritimus*) consume large quantities of high trophic marine mammals such as seals, whose blubber contains OHCs contaminants [160]. This effects their metabolism and reproductive cycle [160].

#### 3.1.4. The Environmental Impact of Emerging Materials

The nanotechnology industry is expected to exceed USD 125 billion by 2024 [194]. The environmental, safety, health and sustainability (ESH/S) impacts of emerging materials such as engineered nanomaterials (ENMs) need to be assessed [195]. ENMs are a bottom-up nanotechnology in which nano-surfaces, particles and fibres are synthesised from molecules [196]. The environmental impact of ENM depends on how the ENMs are processed from raw materials, how they are incorporated into a final nanoproduct, in which surroundings this final product functions, and how it is eventually disposed of [197]. The rapid development of nanoparticles (NPs) has led to an augmented complexity and diversity of materials, and regulation and predictions of environmental impacts have not been able to keep up [194]. The use of semiconductor nanomaterials (NMs) has increased the processing power of mobile devices. This is due to wide band gaps of semiconductor NM in electronics and chipset use [198]. The semiconductor industry must address the ESH/S threats of using these materials in their products. Validated methods of toxicological studies have yet to be developed. Some published reports do focus on the toxicity of pristine nanomaterials but fail to quantify the risk after release into the environment [199].

#### 3.1.5. Developing Universally Accepted Guidelines for Assessing ESH/S Challenges

The semiconductor industry is a dynamic industry of continual technological innovation and change. New technological advancements change production processes and so generate more types of by-products or wastes [32]. Companies open and close and equipment use changes. These ever-evolving dynamics make it challenging to accurately assess environmental health and safety concerns in the industry [33]. It is also hard to obtain information on the number and volume of chemicals used in the actual semiconductor factories [200]. To address ESH/S challenges, it is important to understand the actual chemical usage in the industry. There is a need for methodologies to define and measure sustainability by both technology generation as well as at the factory infrastructure level [56]. 

However, a universally accepted or applicable strategy for assessing ESH/S currently does not exist. There are no clear guidelines or standards for how frameworks, methods and tools can be applied to select the least ESH/S impactful materials [33]. 

**Figure 6:**
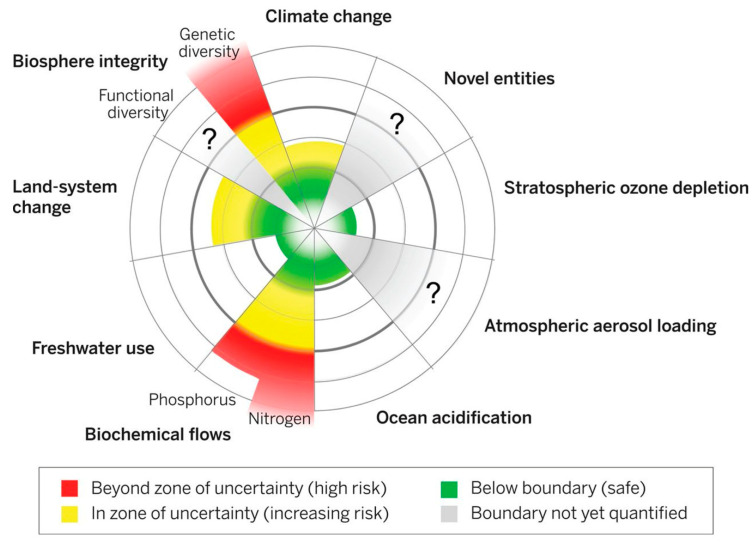
Current status of planetary boundaries control variables [26]. The control variables describe the different anthropogenic perturbations from stable Earth systems. Different levels of risk range from safe, to not yet quantified or increasing, to high risk. The intersection of the below boundary (safe) and in zone of uncertainty (increasing risk) defines the planetary boundary. From [26]. Reprinted with permission from AAAS.

Avoiding irreversible effects of chemicals on Earth systems requires a change in how environmental challenges are assessed and prevented. Yet, there is no fully agreed-upon method for global analysis of chemical pollution [201]. It is the responsibility of all researchers involved in industrial development to operate in the safe zone of the planetary boundary framework [195]. This means operating in the safe zones in Figure 6 below. Figure 6 combines scientific understanding of ES functioning with precautionary principles to define levels of anthropogenic perturbations that are sufficiently low enough to avoid risk of destabilisation of the ES [26]. Many of the techniques in semiconductor research are novel and operate in zones of uncertainty and not in the green zones - this is a cause of concern [196].

### 3.2. Green Chemistry and Environmental Sustainability in the Semiconductor Industry

So far, the use of BCP and brush lithography techniques in the semiconductor fabrication process have been discussed. Some of the possible sustainability advantages of ASD and DSA have been mentioned, but how can these techniques be integrated into the semiconductor industry to tackle sustainability challenges, and help meet environmental regulations governing the electronics life cycle?

**Figure 7:**
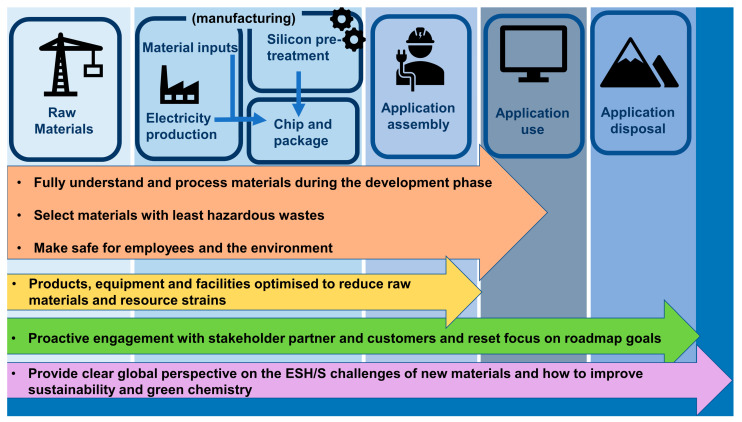
The six core ESH/S roadmap strategies [202]. This figure shows which stages of the microelectronic life cycle [46] these environmental roadmap strategies can influence.

The International Roadmap for Devices and Systems (IRDS) Factory Integration 2020 edition identifies six core ESH/S roadmap strategies [202]. These strategies are illustrated in Figure 7. BCP and polymer brush nanofabrication routes must support these ESH/S strategies.

#### 3.2.1. Selecting Materials with the Least Hazardous Waste

Green chemistry involves selecting chemicals that are non-depleting, non-toxic and non-persistent in the environment [203]. Emerging technologies may provide alternative greener fabrication processes and improved capability to select raw materials with reduced hazardous waste [204]. For example, pollutants from the incineration of photoresists during the waste treatment phase may be reduced or mitigated [184]. Whether the discussed ASD and DSA techniques present such an opportunity has yet to be determined.

#### 3.2.2. Fully Understand the Materials and Process during the Development Phase

Materials used should have a quantified risk, and the molecular mechanisms by which molecules interact with their environment and living organism once released should be understood [26,194]. Models should be developed to quantify possible environmental toxicity and minimise them [191]. Applying LCA methodologies at this early stage of development is crucial to optimising green chemistry methodologies and environmental sustainability considerations [41]. LCA helps ensure that the least hazardous molecular mechanisms are selected whist still preserving product functionality. Additionally, the use of LCAs facilitates informed decision that balances long-term environmental risks and production costs [45,191]. Furthermore, new regulations and natural resource constraints may lead to delays and unforeseeable increases in costs [44,45]. A method for assessing the molecular mechanisms of waste products for this alternative technique can be achieved using LCA [205].

#### 3.2.3. Factory and Industry Supply Chain Should Be Made Safe for Employees and the Environment 

Little is known about the exact chemical nature of the substances that factory workers are exposed to because of the secretive nature of trade agreements concerned with processing technologies [29,34,206,207]. BCP and brush lithographic methods can use non-toxic materials to facilitate deposition, for example, the use of green solvents and biodegradable and biocompatible polymers as discussed previously. BCP and brush nanofabrication techniques may facilitate the increased use non-toxic materials. However further research is required to determine if this is the case. 

#### 3.2.4. Selecting Products, Equipment and Facilities Optimised to Reduce Raw Materials and Resource Strains

**Figure 8:**
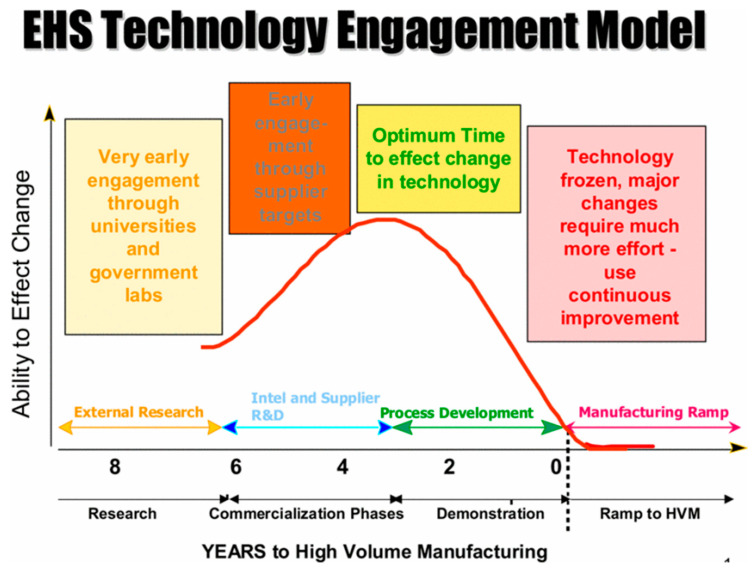
Environment, Health and safety (EHS) Technology Engagement Model [40]. This figure emphasises how far ahead manufacturing ramp engineers and designers must work to integrate sustainable practices into new technological innovations [40]. © [2008] IEEE. Reprinted, with permission, from [40].

Humanities management of raw materials and resources requires deep transformation if we are to avoid crossing the planetary boundary threshold [208]. Implementing green chemistry methodologies early on in the design phase improves the effectiveness of decision making when responding to environmental regulation, and resource constraints [45]. Figure 8 illustrates when the integration of sustainable practices into new technological innovations is most effective [40]. ASD and DSA techniques for bottom-up lithography are in the early design phases. Integrating green chemistry and environmental considerations during this phase may help reduce raw material and resource constraints.

The semiconductor industry faces a change in global sustainability regulations and pressure to reduce the amount of greenhouse gas emissions [27]. Brush and BCP lithography may provide opportunities for the development of new facilities with reduced raw materials and resource strains.

#### 3.2.5. Proactive Engagement with Stakeholders, Partners and Customers and Reset Focus on Roadmap Goals

Nanoscale fabrication relying on DSA of BCPs or ASD of polymer brushes is recognised as a valuable platform for the next-generation of functional structures with the added benefit of potential environmental sustainability [209]. It cannot be emphasised enough that communicating with stakeholders, partners and customers to inform them of improved environmental sustainability in manufacturing is essential to achieve environmental sustainability roadmap goals [33]. Stakeholders may exert pressure on a company’s efforts at green management through ever-increasingly stricter environmental regulations and awareness of environmental issues [44,210]. Profits can be affected by a company’s environmental image as customers would be swayed to buy greener products from more environmentally conscious companies [211]. From an economic perspective, nanotechnology is expected to become a key pillar for the European economy [44]. It is vital to foster an open culture of informing about and communicating identified hazards with customers in order to avoid damage to industry through socio-economic impacts [196]. Using bottom-up lithographic processes may help satisfy these pressures, providing the technique offers an improved environmentally sustainable manufacturing route. Engagement with customers and stakeholders may encourage more intensive research and development to determine if the techniques can be developed as a sustainable alternative to current lithographic techniques. 

#### 3.2.6. Provide a Clear Global Perspective Concerning the ESH/S Challenges of New Materials, and How to Improve Sustainability and Green Chemistry

There are many challenges to overcome to provide a clear methodological approach to assess and tackle ESH/S challenges in the semiconductor industry [207]. The final section of this paper outlines how the LCA of emerging manufacturing processes such as bottom-up lithography can be used to address ESH/S challenges and improve green chemistry practices.

### 3.3. Are There Environmental Incentives to Pursue the Alternative Lithography Strategies?

The ever-increasing use of electronic devices in all sectors of the economy is affecting the environment. Efforts to improve and mitigate environmental risks and impacts during lithographic processes are necessary [46]. Lithographic processes are highly toxic and have been shown to have serious health effects on workers such as higher risk for spontaneous abortions for those working in photolithographic process workers than for non-FAB workers [29,149,206,212,213]. The question is how do conventional lithographic processes compare to alternative BCP and polymer brush lithography? To answer this, the sustainability of these techniques in terms of their environmental pollutants is discussed in this subsection and summarised in Table 3.

Carbon emissions: NGL tools such as EUV require high source power. More than 20KW of laser power is needed to produce 200W of EUV power at the intermediate focus due to power loss issues [214]. High power requirements increases strain on resources. The power requirements for polymer brush and BCP lithography are process dependent. Exact power requirements for industrial application of BCP or polymer brush lithography are unknown. However, these techniques do not require complex high-power optical set-ups so have predicted lower power consumption [4,25]. BCP and brush nanofabrication routes as previously discussed have a reduced number of processing steps [4,7,36]. UV exposure of a photomask is not necessary, for example. This may correspond to reduced tool use, energy and resource strain.

Organohalogens: PFCs are used for etching and to clean CVD chambers [157,215]. When under normal operating conditions 10 to 80% of PFCs used in cleaning CVD chambers could pass through the manufacturing tool chambers unreacted and be released into the air [157,179]. This enhances the greenhouse effect [158,215]. When scaling polymer and brush lithography to an industrial scale green fabrication routes can be selected to eliminate or reduce PFC use. For example, using liquid based deposition to replace CVD if PFC are required for cleaning CVD chambers post metal infiltration of BCPs or polymer brushes. The versatility of bottom-up lithography approaches may allow for exclusion of PFCs from initial stages of lithography but may still be required during etching processes. Setups such as vapour deposition chambers or solvent vapour annealing systems currently don’t require use of PFCs [37,124,125,216]. Whether PFC use will be required or can be eliminated for industrial scale polymer brush and BCP lithography requires further research.

Wastewater: High levels of anions and organic pollutants are present in semiconductor industry wastewater [217]. Accumulation of various wastes and by-products in wastewater is of concern [218]. Photolithographic chemicals end up in waste water for example Perfluorooctane sulfonate (PFOS) [219]. Other examples include PFCs in wastewater which contaminate river systems and tetramethylammonium hydroxide (TMAH) from photoresist developer waste water [192,220]. TMAH has high alkalinity, toxicity and is eutrophic to the water environment [221,222]. Polymer brush and BCP lithography do not require Photoresist and photostripper. Future studies should examine the possible impact of polymer brush and BCP nanofabrication routes on wastewater.

Acids, bases, and solvent wastes: During conventional lithography a considerable amount of acid, base and solvent waste is generated. Examples include Isopropyl alcohol (IPA) and acetone required for the prevention of photoresist build up during spin coating [157], Ethylbenzene and Ethylene Glycol Ethers are used in lithography [223,224]. These chemicals are of environmental health and safety concern and cause issues such as Reproductive toxicity embryonic and developmental toxicity with exposures to low airborne concentrations [212,213,224]. Tetrachloroethylene is used in lithography in fabrication (FAB) and can cause Cervix, oesophagus, non-Hodgkin lymphoma [223]. Polymer brush and BCP lithography also makes use of acids, bases and solvents. For example toluene and tetrahydrofuran (THF), but also green solvents such as water, ethanol, or ethyl lactate [113,114,115,116]. Whether the acid, base and solvent waste from conventional lithography is more harmful than that from BCP and polymer brush lithography requires further research. However the potential use of chemicals with reduced toxicity and use of green solvents is promising [179]. The versatility of nanofabrication using BCP and polymer brushes means that green fabrication routes can be incorporated into lithographic techniques for example CVD can be used instead of spin coating and reduce material waste [104]. In conventional lithography the pattern in resist layer is transferred to underlying substrate via techniques such as etching [225]. Wet etching processes use various acids and bases for example Sulfuric acid. Sulfuric acid can lead to lung and laryngeal cancer [223]. Etching processes are still used in bottom up process [58,104,226]. Technique used depends on polymer choice. Perhaps number of polymers available and techniques to remove polymer post etch will allow for selection of a green fabrication route.

Photoresists and photostrippers: Wastes from photoresists and photostrippers are persistent, bio accumulative, and toxic, and thus of substantial environmental concern [180,219,227]. photolithographic chemicals such as PFOS add to the toxicity of waste water [219]. Other chemicals of concern include Developer tetramethylammonium hydroxide which is toxic to marine life [228,229]. Photoacid generators (PAGs) are widely applied in photolithographic processes [230,231] There associated environmental toxicity, persistence, and bioaccumulation is of concern [230]. Photoresists and photostrippers are not required for BCP and polymer brush nanofabrication routes. Photoresists after exposure to light decomposes into harmful chemicals [149]. Whether the associated toxicity of BCPs and polymer brushes post pattern removal requires is more or less harmful than currently used photoresists quantification. The reduced number of steps and avoidance of UV exposure and photomasks however suggests a possibly more efficient process with reduced environmental toxicity. It is also worth considering that precise environmental impact of photoresists and photostripper is challenging to determine as a result of trade secrets [200]. Thus the exact environmental threat of photoresist and photostripper compared to materials used in BCP and polymer brush lithography may be challenging to determine.

BCP and polymer brush wastes: BCP and polymer brushes are removed via processes such as reactive ion etch or UV ozone [125,226]. Photoresists, after exposure to light, decompose to low–molecular weight compounds, such as phenol, cresol, benzene, toluene, xylene, and other benzene-based aromatic compounds, in addition to the carrier solvents [149]. Further research is required to determine if the removal of polymer brushes and BCPs post pattern transfer is more or less harmful than conventional lithographic techniques that use photoresist. Reduced etch steps have been reported for DSA which suggests possible reduced environmental pollution from wastes [232]. Another important consideration is the environmental impact of synthesising polymers [13]. Which raises the question of whether BCP or polymer brush synthesis is more or less harmful than the synthesis of polymers used in top down lithographic processes.

Metals: Trace elements Gallium (Ga) and Indium (In) are used for thin film growth, photolithography, and polishing [182]. Ga and In have reported toxicity as high as heavy metals and are deposited during lithography [183]. Additionally, the accumulation and use of heavy metals in the semiconductor industry is damaging to aquatic ecosystems and is hazardous for humans and wildlife [233,234,235]. Both top-down or bottom up lithographic processes pattern metals on substates. However, an interesting question for further research is which process is most efficient and wastes the least amount of metal precursors.

The findings of this review suggest that there are environmental incentives to pursue alternative lithography strategies. This conclusion is based upon the following reasoning: Some of these environmental incentives include: The versatility of bottom-up lithography methodologies is an asset that other techniques restricted by optical resolution don’t have. This versatility may give a greater number of options for finding a green fabrication route.When scaling nanofabrication techniques from lab scale to industrial scale green chemistry principles can be used to devise the most sustainable fabrication route [179]. Polymer brush and BCPs don’t require steps such as soft bake, alignment and exposure, development and hard bake that are required in conventional lithography [227]. This may reduce material and energy waste. ASD and DSA techniques represent an opportunity to reduce the generation of pollutants and waste. The economic advantages of these techniques have been reported [4,36,232]. Further research is required however to determine exactly how advantageous bottom lithography techniques are environmentally and how feasible their implementation in industry is. A structured investigation that considers the complexities of integrated industrial systems and ecosystems is required [236]. We outline an LCA approach to achieve this in the next section.

## 4. Life Cycle Analysis Framework to Assess the Viability of Green Lithographic Strategies

**Figure 9:**
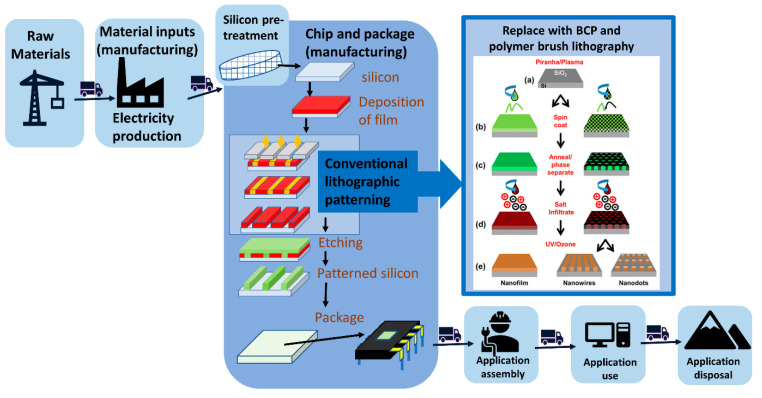
The semiconductor life cycle and the stage in which BCP and brush lithography could be implemented in the life cycle. The stages of the process are adapted from Figure 5: The general phases in a component life cycle [46]. Emerging lithographic sages are adapted from Figure 4 [18].

LCA offers a convenient tool to guide selection and implementation of novel methods for industries. The term LCA is a scientific methodology developed during the 1990s which allows a wide range of specific environmental impacts of a product from cradle to grave to be evaluated in a study [38,48,237,238,239,240]. The semiconductor life cycle is modified in Figure 9 to include brush and BCP lithography. The purpose of this section is to develop a potential framework for how LCA of BCP and polymer brush lithography might be performed. Application of this framework requires data detailing energy consumption, efficiency and wastes of lithographic process by semiconductor industries. Additionally, it requires data detailing BCP and polymer brush lithographic processes at the industrial and developmental stages. The conclusions for such an LCA analysis allow for quantification of how environmentally and economically beneficial BCP and polymer brush lithographic techniques are. 

To perform an LCA, a complete understanding of the life cycle of a product is generally required [241,242]. Thus, in order to extrapolate a framework for LCA analysis of BCPs and polymer brush lithography in the semiconductor industry ex-ante LCA is required [241,243,244]. Ex-ante LCA examines the projected performance of a product or technology during the early stages of production and before commercial deployment on a large scale [241,245]. Most LCA studies of this nature conclude that new insights during the development of new technologies can support policy makers in their work [245,246]. In this review we adopt the broadest definition of ex-ante LCA by including in its definition prospective LCAs or anticipatory LCAs as is done by Buyle et al. [242].

Ex-ante LCA analysis cannot solely follow the methodology described in the LCA manuals. This is because they typically provide guidance on modelling and assessing environmental impacts after products have been in commercial use for a sufficient amount of time for data to become available on their performance [244]. Thus LCA of an emerging technology comes with challenges such as the lack of a clear definition of a future system or the absence of comparability, the presence of uncertainties, and the inability to predict for an upscaled system using the available scarce LCA data [243,245,247]. Overcoming these challenges at the early stages of research and development facilitates the reduction of environmental burdens and allows for process optimisation before production on an industrial scale [243,248]. Here we detail the stages of LCA required for an evaluation of emerging BCP and polymer brush lithographic methods. During the Inventory analysis stage, we discuss ex-ante LCA principles required for evaluation of BCP and polymer brush lithography techniques.

**Figure 10:**
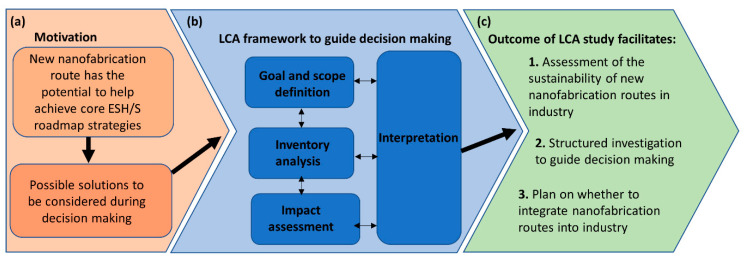
Objectives and phases of polymer brush and BCP lithography LCA. The arrows indicate the progression from part (**a**) initial Motivation to (**b**) building a LCA framework to guide decision making to part (**c**) the outcome of the LCA study. In part (**b**) the four stages of the LCA are outlined as defined by the International Organization for Standardization (ISO) 14000 standards — specifically, 14040 and 14044 [237,250].

Figure 10 summarises the objectives and phases of polymer-based lithography LCA. Part A of Figure 10 displays how BCP and brush nanomanufacturing routes have the potential to help achieve core ESH/S roadmap strategies. The application of the LCA framework as a guide to decision making is outlined in part B. Life cycle assessment is a stepwise method comprising of: (i) goal definition and scope definition, (ii) life cycle inventory analysis, (iii) life cycle assessment and (iv) interpretation [249]. LCAs are iterative: thus, if the framework in this review is applied, the LCA process should be continually refined as the study is carried out [205]. Part C in Figure 10 illustrates some of the possible outcomes of such a study.

### 4.1. Goal Definition and Scoping

In the LCA framework the first step is often the goal definition and scoping [38]. This step involves outlining the type of LCA that will be applied, the objective of the study and the definition of system boundaries [49]. The first stage is to define the reasons for carrying out the study [251]. In this case, it is to determine if ASD and DSA deposition methods can provide a sustainable and financially viable nanofabrication route. This in turn provides decision-making support when determining future investments and environmentally sustainable development of the technology and industrial integration [240]. Whether the results are intended for public discloser and who the target audience is, the commissioner of the study and other influential actors, should be stated [205]. It is also valuable to consider if any limitations due to chosen methodology or assumptions affect the usability of the LCA results [252]. For example, ASD and DSA deposition techniques are at research and developmental stages and assumptions may be made during LCA due to lack of data on their industrial performance.

The specific reasons for carrying out the study should be re-stated at this stage and it should be clear as to whether the study is comparative or non-comparative [240]. For a comparative LCA, the objective is to compare emerging bottom-up lithographic technologies and conventional top-down technologies. The level of the temporal development and the technological development must be aligned to the same development stage for all modelled technologies [245]. For polymer brush and BCP techniques the time at which the comparison is made between it and the conventional lithographic technique it is replacing should be the same. This is because both techniques should be compared at the same technology readiness level (TRL) and manufacturing readiness level (MRL) [242,245]. For non-comparative ex-ante LCA of emerging bottom-up lithography, the aim is to reduce environmental impacts during the design and development phase [245]. 

The function of the system being analysed is stated at this stage [205]. As previously discussed ASD and DSA are both pattern transfer technologies. The function of DSA of BCPs is to self-assemble nanodomains for nanoscale patterning of a substate [253]. For ASD the function is to use a polymer brush film to deposit material in one region of a substrate selectively while simultaneously avoiding deposition in another region [21,254]. A functional unit is also defined as it enables comparison of two essentially different systems and provides a basis for defining all inputs and outputs into the system [246,254]. The functional unit is described in terms of some quantifiable properties of the system under consideration [205,251]. An example of a functional unit from the work of Schmidt et al. is defining inputs and outputs as per square meter of fabricated dynamic random access memory (DRAM) or logic wafers of thickness 0.75 mm [47]. This functional unit then provides a basis for describing the system’s reference flow. An example from the same study are gaseous chemical waste emissions released in kg per hour per square meter output of processed DRAM wafer [47]. In the case of DSA and ASD technologies a functional unit could be defined as chemical mass (kg) or energy (J) required per square metre to produce a patterned silicon wafer. Functional units should incorporate the margins of error and clearly defined data uncertainties [245,255,256].

**Figure 11:**
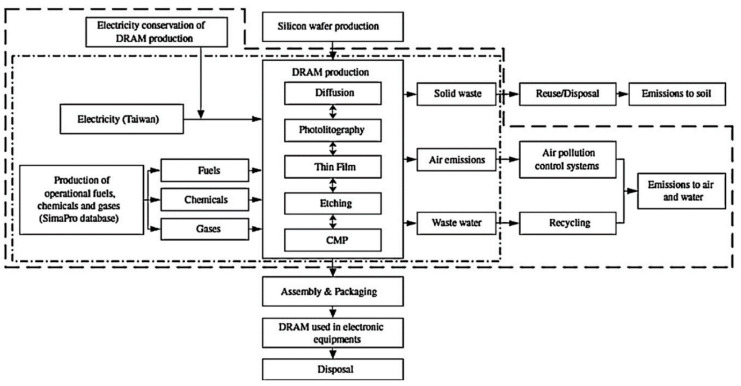
Production of DRAM divided into three process modules from which system boundaries of the LCA study can be selected [49]. Energy and Buildings, Volume 56, Shih-Cheng Hu, Angus Shiue, Hsien-Chou Chuang, Tengfang Xu, Life cycle assessment of high-technology buildings: Energy consumption and associated environmental impacts of wafer fabrication plants, Pages 126–133, Copyright (2013), with permission from Elsevier [49].

Followingly, the system boundaries are outlined, detailing how much of the life cycle will be examined [252,257]. For example, Shih-Cheng Hu et al. divided the production of DRAM into separate process modules [49]. The system boundaries for LCA of DRAM wafer processing in a fab are then selected by choosing which system modules are within the boundaries of the analysis (the dashed lines in Figure 11) [49]. The choice of the system boundary may affect how an emerging technology such as ASD or DSA of polymers ranks when compared to the conventional lithographic techniques, leading to misinformed decisions [258].

The systems boundaries for the mature technology in this review would be those defined for the production of an integrated circuit [181]. Therefore, the process modules would be: raw wafer manufacturing, wafer processing, assembly and packaging. The process module relevant to the LCA comparing emerging deposition techniques to conventional ones is wafer processing. At this stage, nanostructures are created on substrates using conventional lithographic techniques [181]. Less relevant processes and elementary flows can be either cut off or estimated. For example, investments in raw wafer manufacturing are irrelevant to the objective of the study, and can be defined outside the system boundary thus the data can be excluded. To complete the initial round of scope, a definition of a basis for life cycle inventory analysis (LCI) needs to be established [205,252]. The derived data quality needs to be outlined, information sources must be identified and plan reporting and reviewing have to be determined. More information on scope definition can be found in LCA handbooks such as the ILCD handbook [205].

### 4.2. Life Cycle Inventory Analysis

The next stage in the LCA framework is LCI within system boundaries [49]. LCI is the quantification of raw material and fuel inputs into a system as well as the output of solid, liquid and gaseous emissions from a product system [49]. Table 4 gives some examples of the kind of data required for inventory analysis

#### 4.2.1. Data Collection for the Mature Technology

To structure and guide the data collection process, a questionnaire can be made to outline what data is required for LCA. An example can be found in the work by Schischke et al., where they outline a questionnaire to guide the process of data collection for employees of the fab and infrastructure facilities in collecting the data [181]. Chemical information provided by a company for the mature deposition technology data set could include chemical information on the phase, product number, factory, chemical product (e.g., photoresist), ingredients, chemical abstract service (CAS) number, processes used, number of units and the pattern of usage [200].

Collected data should be presented in terms of per reference flows to make comparisons possible [181]. The methods chosen for LCI comparisons depend on what data is available and how much must be quantified using computational approaches [259]. Possible barriers to LCI may include difficulty accessing reliable and verifiable data from manufacturers that details process-specific emissions, chemical inputs and outputs required [260].

#### 4.2.2. Data Collection for the Emerging Technology

**Figure 12:**
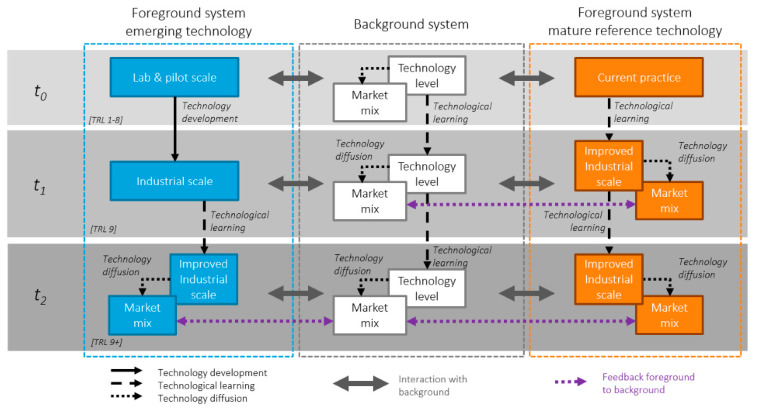
General framework for ex ante life cycle assessment. Blue signifies evolution of emerging technology under study, grey dashed box signifies the background system and orange signifies the foreground system of the mature technology. Reprinted, with permission, from [242].

A recent framework for ex-ante LCA by Buyle et al. is shown in Figure 12. t_0_ refers to the current situation, t_1_ refers to the moment when the emerging technology reaches maturity and t_2_ refers to when the technology has been implemented for a while. Foreground data is collected to describe and model the emerging and mature technology [244]. Background data usually consists of data for upstream supply chains necessary for emerging and mature technologies to perform functions such as socio-economic ones [244].

#### 4.2.3. Scaling to t_1_—Industrial Scale

BCP and brush nanomanufacturing routes fall into the emerging technologies bracket shown in blue in the above diagram. t_0_ is the first stage of technological development TRL, where the technology is not yet available commercially and is not being developed at an industrial scale [242,245]. The first challenge is to explore how BCP and brush lithography techniques will operate at a later industrial scale (high TRL) at time t_1_ [242,245]. This involves the scale-up of a new technology from lab-to-fab using an inventory of existing production processes but with novel techniques replacing existing ones [242]. In order to do this, ex-ante LCA must deal with unknown situations and make predictions despite possible lack of information on novel processes, impacts of other industries, socio-economic evolutions, etc [242]. This may result, for example, in data quality decreasing with upscaling [245]. 

Piccinno et al. describe a 5-step process for scaling-up lab processes to industrial scale processes for LCA [248]. Following this methodology, in stage one, a laboratory protocol publication or a patent document outlines brush and BCP nanofabrication methods. This information is then used for a pilot scale technology. The laboratory protocol in stage two is used to develop a BCP and brush nanofabrication processes strategy that could be scaled-up to an industrial process. The scale, apparatus and input materials should be described in detail. Each individual process is then scaled up. In stage three the links between the individual processes are included. Finally in stage four an industrial scaled up process is achieved and evaluated using LCA [248].

#### 4.2.4. Scaling to t_2_—Improved Industrial Scale and Market Mix

The next question is whether the emerging technology with unresolved pathways is likely to outperform current technologies. After emerging brush and BCP nanofabrication methods are used to develop semiconductors at an industrial scale, the field will continue to evolve, and improvements will be made from research and development. The market share will not be stagnant. Thus, the second challenge is to estimate the technological challenges and market share at stage t_2_ [242]. To what degree will emerging technologies succeed or fail at outperforming existing technologies? Strategies such as probabilistic model with global sensitivity analysis (GSA) can be used to assess the sustainability of the emerging product [43].

#### 4.2.5. Quality of Data for Emerging and Mature Technologies

Assumptions are made to extrapolate laboratory processes to large-scale industrial processes [242]. This is because there is no commercial production of semiconductors processed by BCP and brush lithography techniques.

There is also a limited availability of primary manufacturing data for various semiconductor types from the LCA literature which itself also has a limited scope [261]. For example, a study by Lim et al. on the chemical usage in the semiconductor industry could not detail all the hazardous chemicals in the LCI because of trade secrets in the chemical information data base (DB) provided by the company [200]. This data is essential to assess the present impact on environment and maintain future ecological hazards at a negligible level while fostering a high level of industrial and socio-economic development. 

The type of the LCI data basis constructed depends on the quality, quantity and the kind of data collected [259]. LCA is an iterative process; the first LCI will develop an initial life cycle that can be used to calculate initial LCI data basis which might require future refinements.

### 4.3. Life Cycle Impact Assessment

Life cycle impact assessment (LCIA) considers human health, the natural environment, and natural resource usage issues [262]. Guidelines for assessing possible impact categories are detailed in LCA handbooks such as the ILCD handbook and ISO standards such as ISO14040 [205,262]. Many methodologies and LCA modelling tools are available to perform an environmental analysis of a product or a service system [49,262]. The LCIA should be reviewed once it’s completed and the LCA process should be revised and improved to identify any analytical or methodological limitations or errors [205]. An example of an impact classification scheme used in an LCIA is shown in Table 5. This system allows for classification of most impactful substances and resources in terms of pollution caused during the production of a functional unit [30]. Table 5 shows that this multi-criteria analysis looks at different environmental impacts or impact categories. ISO 14044 states that for most LCA studies existing impact categories, category indicators or characterisation models will be selected.

These impact factors are then quantified by impact category indicators [262]. However, these impact categories commonly described in the LCA handbooks are insufficient to fully describe the impacts of emerging technologies [244]. When describing the impact of BCP and brush lithography techniques, care must be taken to recognise that the novel technology may display unexpected new impacts not fully described in LCIA. Additionally, the characterisation factors relevant for the emerging technology may change over time [244].

### 4.4. Interpretation

The final stage of LCA is to interpret all the findings, and to quantify and evaluate them before making conclusions and future recommendations [248]. LCA modelling tools are software can be used to intemperate the results [263]. Knowledge transfer issues in the semiconductor industry may be a barrier to progression in the field of LCA and environmental sustainability [40]. Collaboration between research and academia to develop standardized data collection methods, robust datasets, a coordination across the supply chain and international agreement on how to address environmental sustainability concerns in the semiconductor industry is crucial [40]. EHS technological engagement with universities, government labs and supplier targets before process development and manufacturing is essential when integrating environmental sustainability into new technological innovations in the semiconductor industry [40].

## 5. Conclusions

The complexities associated with continued miniaturisation of ICs have created opportunities for alternative lithographic approaches [6]. Lithography is a cornerstone of the semiconductor manufacturing industry [154]. For it to continue to support cost effective solutions for semiconductor manufacturing, environmental sustainability must be addressed. There is an ever growing focus on producing equipment and facilities that consume less raw materials, create less waste and consume less power in the microelectronics industry [202]. Both the cost and the environmental sustainability of lithographic processes must be considered when developing patterning technologies.

Potentially, polymer brush and BCP lithography could be implemented into industrial semiconductor manufacturing. These bottom up lithographic techniques may provide cost-effective and environmentally sustainable alternative nanofabrication routes. LCA is suggested as a means for future studies to the evaluate environmental sustainability of BCP and polymer brush techniques before the technology is implemented on an industrial scale. To the extent of our knowledge, this review is the first time in the history of the semiconductor industry that a LCA of an emerging lithographic technique recommended before implementation on an industrial scale. It was found that despite the high volume of literature on BCP and brush lithographic methods, their potential environmental impact if integrated into industry has yet to be evaluated. There are economic and environmental incentives to pursue these alternative lithographic strategies. However, the magnitude of how environmentally and economically beneficial they are is unknown. Future studies could use the proposed LCA framework to quantify the environmental and economic benefit of BCP and polymer brush lithography on an industrial scale and thus provide more conclusive support for their implementation in industry.

LCA facilitates the transition from core ESH/S roadmap strategies and ideas to facilitate informed decision-making before the final implementation of new technologies in the industry. The fifth core roadmap strategy suggests that proactive engagement with stakeholder partners and customers is vital for the development of these nanofabrication techniques from research to industrial implementation. LCA offers a basis to make this possible, by having a standardised means of communicating credible information about the environmental performance of the products [39,236]. LCA in the semiconductor industry is challenging; trade secrets and patents make it hard to obtain information on the chemical content, ingredients, CAS number and information on the hazards these chemicals pose [200]. In addition to this, the industry has been changing rapidly over the last few years and with it the environmental, safety and health risks it poses [29].

It is important for the future of sustainable development of nanotechnology that LCA is performed at the manufacturing stage [35]. The environmental burden of nanomanufacturing techniques should be considered as they are a major contribution to the life cycle impacts of the nanoproducts they create [35]. Studies of this nature are imperative for the development of new technologies and manufacturing routes, not just for the semiconductor industry but for all industries [35]. Thus, this review offers a unique perspective on how researchers can engage in environmental stewardship by evaluating and improving the environmental sustainability of new technologies before they reach an industrial scale.

## Figures and Tables

**Table 1:** A comparison of various lithographic techniques.

Category	DSA of BCPs	ASD of Polymer Brushes	EUV Lithography	Nano-Imprint Lithography	Optical Lithography
Principle of operation	Microphase separation produces self-assembled architectures [15]. Structural modulation by selection of molecular composition and weight [12,125].	Polymer brush’s metal binning sites facilitate deposition of inorganic films [18]. Thickness controlled by SVA and deposition process [124].	Relies on a wavelength of 13.5 nm instead of 193 nm used in conventional optical lithography [144]. Principle of operation relies on reflection [51].	Mask physically displaces photoresist to pattern it before cross linking [144,171]. Once resist is cured, mould is removed and patterned resist is used for manufacturing [171].	UV light and mask to transfer patterns. photosensitive substrate selectively exposed. Reactive ion etch transfers the pattern to the substrate [169].
Efficiency and energetic costs	Varies. Industrial SVA system requires development with fast, precise wafer scale processing [123,124,125].	Grafting of polymer brushes to substrates in seconds [18]. Requires standardised industrial process.	Recent EUV tools have productive capacity of 125 wafers per hour [164]. Hot plasma 20-50 eV or accelerator [154]. technology is energetically expensive [16,154].	Capable of producing over 40 wafers of 300 nm per hour with a defect rate less than 9 pcs/cm^2^ [171].	Short time for pattern. E.g., 150-300-mm wafers per hour and 40-nm two-dimensional pattern resolution of a scanner with pixel throughput of 1.8T pixels per second approximately [144].
	low-temperature vapor phase deposition process cuts costs and improves environmental sustainability [21,137].				
Wastes generated	Solvents, excess inorganic precursor for metal infiltration, excess polymer during deposition stage.		Solvents are typically volatile and flammable. Polymer matrix, photoactive compounds and cross-linkers in polymer photoresist are toxic and non-degradable [149,172].		
Tool complexity	Thickness and pattern formation control can be achieved with simple chambers consisting of temperature and gas flow systems [124,125]. Predicted low cost of ownership. These techniques do not require the use of photoresists and avoids acquisition costs.		Expensive and complex multilayer optics, hot plasma or acceleration technology and high vacuum. Cost of tool > $30 million [137,144,154].	Depends: TE-NIL uses heat and pressure. SFIL uses capillary forces, pressure, and light exposure. Improved tool performance required [173,174,176].	Acquisition cost of photolithographic resist coat system is $950,000 [139].
Disadvantages	High defect density of BCPs [138]. Poor etch contrast of polymer blocks [14].	Improved range of metals that can be deposited [18].	Obtaining high reflectivity [144], improved etch and deposition defect mitigation and repair of mask, installation and power costs [16,142,162,166,170].	Needs reduced process steps and improved mould material, fabrication quality, mass production capacity, reduced overlay and defectivity [16,33,171].	Photolithography can no longer be further optimised as it has an intrinsic resolution limit [51,137].
	Standardised methodology and further research required [7,138].				
Advantages	No diffraction limit in resolution [137], directly pattern functional materials [137], efficient for 3D patterning [137].	Reduction of processing steps [133]. self-aligned patterning with capability of extending to 3D [17].	Improved: cycle time, increased number of patterning levels, line edge roughness, high etch resistance, increased sensitivity [33,138].	Facilitates change in flash memory from scaling horizontally to vertically. Simple processing steps, high throughput, low cost and high resolution [51].	Established technology being the dominate patterning technique since the beginning of IC production [51].

**Table 2:** Comparison of size and resolution of resulting nanofabricated structures.

Category	DSA of BCPs	ASD of Polymer Brushes	EUV Lithography	Nano-Imprint Lithography	Optical Lithography
Accuracy and Resolution	Needs research and development for: Pattern features control, Defects minimisation [138], Defect mitigation and repair, and techniques [123,124].		Stochastic issues leading to critical dimension variation, critical pattern defects and feature roughness [33,170].	Limited by size of features on mould. high fidelity of pattern transfer [137]. Defect density and mask damage issues [137]. Suitable material for moulds [171,175].	Resolution improvements reaching limits [52,144,154,155].
Size	BCP patterns 5 to 200 nm size range [123] with dot, line hole or lamellar patterns [126]. May improve feature control to sub 7 nm and pitch multiplication [138].	Depends on the chemistry of the end-functional group and polymer deposition technique used.	Main candidate for sub10nm manufacturing [51,170]. 25 nm 1:1 line/space resolution with an approximate 2.7 nm line edge roughness [162].	Allows for patterning features of sub-100 nm possibly with features as small as 10 nm [146,174].	Smallest features approximately equal to wavelength of light used [52]. Wavelength of 193 nm or longer is generally used [144,177].
Predicted or used in HMV in device type	3 nm node logic [16].	unknown	1.5–7 nm node logic [16].	Device type: 3D Flash Memory [16].	Limited by wavelength.

**Table 3:** Environmental impact of conventional lithographic routes is directly compared to emerging BCP and polymer brush lithography.

Sustainability Issue	Conventional Lithography		Bottom-Up BCP and Polymer Brush Nanofabrication Routes	
	Process	Impact	Process	Impact
Carbon emissions	NGL tools such as EUV require high source power [214].	High power requirements increased strain on resources.	Predicted lower power requirements due to high-power optical set ups not being necessary and reduced number of processing steps [4,18].	Predicted reduced power consumption costs and energy resource strain.
Organohalogens	Use of PFCs for etching and to clean CVD chambers [157,179,215].	Enhances greenhouse effect [158,215].	Versatility of bottom-up lithography may allow for exclusion of PFCs from initial stages of lithography but may still be required during etching processes.	When scaling to industrial-scale green fabrication route can be selected. Predicted impact requires further investigation.
Wastewater	Accumulation of various wastes from conventional lithographic processes in wastewater is of concern [192,218,220].	Contamination of water systems, environmental toxicity and eutrophication [221,222,234]	BCP and polymer brush impact on wastewater is point of further research.	Unknown on an industrial scale. LCA studies may help predict impact.
Photoresists, photostrippers, BCP and polymer brush wastes	Wastes from photoresist and photostripper are of concern [34,180,228,230,231]. Photoresists after exposure to light decomposes into harmful chemicals [149]. This step is not required for bottom-up lithography.	Persistent, bio accumulative, toxic and bio accumulative and thus of substantial environmental concern [219,229,230].	BCP and polymer brushes are removed via processes such as reactive ion etch or UV ozone [125,226]. Potential use of biopolymers may avoid harmful by products of etching processes.	Reduced etch steps have been reported for DSA [232]. Further research is required to evaluate environmental impact.
Acids, bases and solvent waste	Wet etching processing uses various acids, bases and solvents of environmental concern for example Sulfuric acid [157,223,234,225].	Health risks and environmental toxicity [212,213,223,224,234].	Acid, base, and solvent use depends on polymer and metals used in BCP and polymer brush lithography.	Unknown environmental impact on industrial scale. Capability of using green fabrication routes [104,179]
Trace elements and heavy metals	Trace elements such as Gallium and Indium have reported toxicity as high as metals and are deposited during lithography [183].	Hazardous for both humans and wildlife [233,234,235].	Metal use is required for lithographic patterning in the electronics industry.	Research required to determined more efficient and less wasteful than conventional lithography.

**Table 4:** Examples of the type of data required for the LCI.

Material and Energy Consumption Used in Raw Materials Extraction/Acquisition, Processing, and Transportation
Material and energy resource consumption during the deposition process
Quantitative data and chemical composition of the wastes: ❖ Wastes that are directly emitted into atmosphere ❖ Wastes that are released into aquatic systems on-sight
Quantitative data on chemical composition of recycled wastes
Qualitive data on chemical wastes disposed of off sight and process of disposal
Transportation emissions for off sight waste disposal

**Table 5:** Distinction between damage and impact categories [30]. Reprinted from [30], with the permission of AIP Publishing.

Damage Category	Impact Category	
Human Health	Human toxicity Respiratory inorganics Photochemical oxidation	Ionising radiations Ozone layer depletion
Ecosystem Quality	Aquatic ecotoxicity Terrestrial ecotoxicology Terrestrial acidification/nitrification	Aquatic acidification Aquatic eutrophication Land occupation
Climate change	Global warming	
Resources	Non-renewable energy	Mineral extraction

## References

[1] Pinto-Gómez C., Pérez-Murano F., Bausells J., Villanueva L.G., Fernández-Regúlez M. (2020). Directed self-assembly of block copolymers for the fabrication of functional devices. Polymers (Basel).

[2] Liddle J.A., Gallatin G.M. (2016). Nanomanufacturing: A Perspective. ACS Nano.

[3] Shalf J. (2020). The future of computing beyond Moore’s Law Subject Areas. Philos. Trans. R. Soc..

[4] Cummins C., Lundy R., Walsh J.J., Ponsinet V., Fleury G., Morris M.A. (2020). Enabling future nanomanufacturing through block copolymer self-assembly: A review. Nano Today.

[5] Sarangan A. (2016). Nanofabrication. Fundamentals and Applications of Nanophotonics.

[6] Black C.T. (2007). Polymer Self-Assembly as a Novel Extension to Optical Lithography. ACS Nano.

[7] Cummins C., Shaw M.T., Morris M.A. (2017). Area Selective Polymer Brush Deposition. Macromol. Rapid Commun..

[8] Cummins C., Gangnaik A., Kelly R.A., Borah D., O’Connell J., Petkov N., Georgiev Y.M., Holmes J.D., Morris M.A. (2015). Aligned silicon nanofins via the directed self-assembly of PS-b-P4VP block copolymer and metal oxide enhanced pattern transfer. Nanoscale.

[9] Paripovic D., Klok H.A. (2011). Polymer brush guided formation of thin gold and palladium/gold bimetallic films. ACS Appl. Mater. Interfaces.

[10] Jeong S.-J., Kim J.Y., Kim B.H., Moon H.-S., Kim S.O. (2013). Directed self-assembly of block copolymers for next generation nanolithography. Mater. Today.

[11] Parsons G.N., Clark R.D. (2020). Area-Selective Deposition: Fundamentals, Applications, and Future Outlook. Chem. Mater..

[12] Cummins C., Alvarez-Fernandez A., Bentaleb A., Hadziioannou G., Ponsinet V., Fleury G. (2020). Strategy for Enhancing Ultrahigh-Molecular-Weight Block Copolymer Chain Mobility to Access Large Period Sizes (>100 nm). Langmuir.

[13] Schacher F.H., Rupar P.A., Manners I. (2012). Functional block copolymers: Nanostructured materials with emerging applications. Angew. Chem. Int. Ed..

[14] Cummins C., Ghoshal T., Holmes J.D., Morris M.A. (2016). Strategies for Inorganic Incorporation using Neat Block Copolymer Thin Films for Etch Mask Function and Nanotechnological Application. Adv. Mater..

[15] Prochukhan N., Selkirk A., Lundy R., Giraud E.C., Ghoshal T., Downing C., Morris M.A. (2021). Large-Area Fabrication of Vertical Silicon Nanotube Arrays via Toroidal Micelle Self-Assembly. Langmuir.

[16] (2020). IRDS International Roadmap For Devices and Systems 2020 Edition Lithography.

[17] Cummins C., Weingärtner T., Morris M.A. (2018). Enabling Large-Area Selective Deposition on Metal-Dielectric Patterns using Polymer Brush Deactivation. J. Phys. Chem. C.

[18] Lundy R., Yadav P., Selkirk A., Mullen E., Ghoshal T., Cummins C., Morris M.A. (2019). Optimizing Polymer Brush Coverage To Develop Highly Coherent Sub-5 nm Oxide Films by Ion Inclusion. Chem. Mater..

[19] Mirzaee-sisan M., Sereshki M., Siadati M.H., Eslami-farsani R. (2019). Metamaterials in the World of Materionics Overview of Fabrication Processes. Int. J. Eng. Sci. Invent..

[20] Chaudhari A., Ghoshal T., Shaw M.T., Cummins C., Borah D., Holmes J.D., Morris M.A. (2014). Formation of sub-7 nm feature size PS-b-P4VP block copolymer structures by solvent vapour process. Adv. Patterning Mater. Process. XXXI.

[21] Lundy R., Yadav P., Prochukhan N., Giraud E.C., O’Mahony T.F., Selkirk A., Mullen E., Conway J., Turner M., Daniels S. (2020). Precise Definition of a “Monolayer Point” in Polymer Brush Films for Fabricating Highly Coherent TiO_2_ Thin Films by Vapor-Phase Infiltration. Langmuir.

[22] Zhao Y., Dunn A., Lin J., Shi D. (2019). Photothermal Effect of Nanomaterials for Efficient Energy Applications. Novel Nanomaterials for Biomedical, Environmental and Energy Applications.

[23] Biswas A., Bayer I.S., Biris A.S., Wang T., Dervishi E., Faupel F. (2012). Advances in top-down and bottom-up surface nanofabrication: Techniques, applications & future prospects. Adv. Colloid Interface Sci..

[24] Teo B.K., Sun X.H. (2006). From top-down to bottom-up to hybrid nanotechnologies: Road to nanodevices. J. Clust. Sci..

[25] Iqbal P., Preece J.A., Mendes P.M. (2012). Nanotechnology: The “Top-Down” and “Bottom-Up” Approaches. Supramol. Chem..

[26] Steffen W., Richardson K., Rockstrom J., Cornell S.E., Fetzer I., Bennett E.M., Biggs R., Carpenter S.R., de Vries W., de Wit C.A. (2015). Planetary boundaries: Guiding human development on a changing planet. Science.

[27] Sueyoshi T., Ryu Y. (2020). Performance Assessment of the Semiconductor Industry: Measured by DEA Environmental Assessment. Energies.

[28] Platzer M.U.S. (2016). Semiconductor Manufacturing: Industry Trends, Global Competition, Federal Policy. Curr. Polit. Econ. U. S. Can. Mex..

[29] Choi S., Yoon C., Kim S., Kim W., Ha K., Jeong J., Kim J., Shin J., Park D. (2018). Comprehensive evaluation of hazardous chemical exposure control system at a semiconductor manufacturing company in South Korea. Int. J. Environ. Res. Public Health.

[30] Matarazzo A., Ingrao C., Clasadonte M.T. (2016). Life Cycle Assessment Applied to the Sector of Microelectronic Devices. AIP Conf. Proc..

[31] Kuo C.H., Hu A.H., Hung L.H., Yang K.T., Wu C.H. (2020). Life cycle impact assessment of semiconductor packaging technologies with emphasis on ball grid array. J. Clean. Prod..

[32] Briones R. (2006). California Semiconductor Industry Hazardous Waste Source Reduction Assesment Report.

[33] (2020). IRDS International Roadmap for Devices and Systems 2020 Edition Executive Summary.

[34] Jang M., Yoon C., Park J., Kwon O. (2019). Evaluation of Hazardous Chemicals with Material Safety Data Sheet and By-products of a Photoresist Used in the Semiconductor-Manufacturing Industry. Saf. Health Work.

[35] Şengül H., Theis T.L., Ghosh S. (2008). Toward Sustainable Nanoproducts. J. Ind. Ecol..

[36] Liu C.-C., Franke E., Mignot Y., Xie R., Yeung C.W., Zhang J., Chi C., Zhang C., Farrell R., Lai K. (2018). Directed self-assembly of block copolymers for 7 nanometre FinFET technology and beyond. Nat. Electron..

[37] Giraud E.C., Mokarian-Tabari P., Toolan D.T.W., Arnold T., Smith A.J., Howse J.R., Topham P.D., Morris M.A. (2018). Highly Ordered Titanium Dioxide Nanostructures via a Simple One-Step Vapor-Inclusion Method in Block Copolymer Films. ACS Appl. Nano Mater..

[38] Nieuwlaar E. (2013). Life Cycle Assessment and Energy Systems. Reference Module in Earth Systems and Environmental Sciences.

[39] Verones F., Bare J., Bulle C., Frischknecht R., Hauschild M., Hellweg S., Henderson A., Jolliet O., Laurent A., Liao X. (2017). LCIA framework and cross-cutting issues guidance within the UNEP-SETAC Life Cycle Initiative. J. Clean. Prod..

[40] Harland J., Reichelt T., Yao M. Environmental sustainability in the semiconductor industry. Proceedings of the 2008 IEEE International Symposium on Electronics and the Environment.

[41] Cucurachi S., Van Der Giesen C., Guinée J. (2018). Ex-ante LCA of emerging technologies. Procedia CIRP.

[42] Boyd S.B., Horvath A., Dornfeld D. (2009). Life-Cycle Energy Demand and Global Warming Potential of Computational Logic. Environ. Sci. Technol..

[43] Vijver M.G., Blanco C.F., Cucurachi S., Guin J.B., Peijnenburg W.J.G.M., Trattnig R., Heijungs R. (2020). Assessing the sustainability of emerging technologies: A probabilistic LCA method applied to advanced photovoltaics. J. Clean. Prod..

[44] Lee K.-H., Kim J.-W. (2011). Integrating Suppliers into Green Product Innovation Development: An Empirical Case Study in the Semiconductor Industry. Bus. Strateg. Environ..

[45] (2017). IRDS International Roadmap For Devices and Systems 2017 Environmental, Safety, Health, and Sustainability.

[46] Villard A., Lelah A., Brissaud D., Villard A., Lelah A., Brissaud D., Villard A., Lelah A., Brissaud D. (2015). Drawing a chip environmental profile: Environmental indicators for the semiconductor industry. J. Clean. Prod..

[47] Schmidt M., Hottenroth H., Schottler M., Fetzer G., Schlüter B. (2012). Life cycle assessment of silicon wafer processingfor microelectronic chips and solar cells. Int. J. Life Cycle Assess..

[48] Higgs T., Cullen M., Yao M., Stewart S. Developing an overall CO_2_ footprint for semiconductor products. Proceedings of the 2009 IEEE International Symposium on Sustainable Systems and Technology.

[49] Hu S.C., Shiue A., Chuang H.C., Xu T. (2013). Life cycle assessment of high-technology buildings: Energy consumption and associated environmental impacts of wafer fabrication plants. Energy Build..

[50] Murphy C.F., Laurent J., Allen D.T. Semiconductor Manufacturing.

[51] Hasan R.M.M., Luo X. (2018). Promising Lithography Techniques for Next-Generation Logic Devices. Nanomanufacturing Metrol..

[52] Harriott L.R. (2001). Limits of lithography. Proc. IEEE.

[53] Mallik A., Vansumere W., Ryckaert J., Mercha A., Horiguchi N., Bömmels J., Zsolt T., Vandenberghe G., Ronse K., Thean A. (2013). The need for EUV lithography at advanced technology for sustainable wafer cost. SPIE Adv. Lithogr..

[54] Hwang B., Huang C., Wu C. (2016). A TOE Approach to Establish a Green Supply Chain Adoption Decision Model in the Semiconductor Industry. Sustainability.

[55] Krishnan N., Boyd S., Somani A., Raoux S., Clark D., Dornfeld D. (2008). A hybrid life cycle inventory of nano-scale semiconductor manufacturing. Environ. Sci. Technol..

[56] Hoefflinger B. (2011). ITRS: The International Technology Roadmap for Semiconductors. Chips 2020.

[57] Nie Z., Kumacheva E. (2008). Patterning surfaces with functional polymers. Nat. Mater..

[58] Mokarian-Tabari P., Senthamaraikannan R., Glynn C., Collins T.W., Cummins C., Nugent D., O’Dwyer C., Morris M.A. (2017). Large Block Copolymer Self-Assembly for Fabrication of Subwavelength Nanostructures for Applications in Optics. Nano Lett..

[59] Kim H.-C., Park S.-M., Hinsberg W.D. (2010). Block Copolymer Based Nanostructures: Materials, Processes, and Applications to Electronics. Chem. Rev..

[60] Brassat K., Kool D., Nallet C.G.A., Lindner J.K.N. (2020). Understanding Film Thickness-Dependent Block Copolymer Self-Assembly by Controlled Polymer Dewetting on Prepatterned Surfaces. Adv. Mater. Interfaces.

[61] Löfstrand A., Svensson J., Wernersson L., Maximov I. (2020). Feature size control using surface reconstruction temperature in block Feature size control using surface reconstruction temperature in block copolymer lithography for InAs nanowire growth. Nanotechnology.

[62] Peng Q., Tseng Y.-C., Darling S.B., Elam J.W. (2011). A Route to Nanoscopic Materials via Sequential Infiltration Synthesis on Block Copolymer Templates. ACS Nano.

[63] Ross C.A., Berggren K.K., Cheng J.Y., Jung Y.S., Chang J.B. (2014). Three-dimensional nanofabrication by block copolymer self-assembly. Adv. Mater..

[64] Poelma J.E., Ono K., Miyajima D., Aida T., Satoh K., Hawker C.J. (2012). Cyclic block copolymers for controlling feature sizes in block copolymer lithography. ACS Nano.

[65] Milner S.T. (1991). Polymer Brushes. Science.

[66] Snelgrove M., Zehe C., Lundy R., Yadav P., Rueff J., O’Connor R., Bogan J., Hughes G., McGlynn E., Morris M. (2019). Surface characterization of poly-2-vinylpyridine—A polymer for area selective deposition techniques. J. Vac. Sci. Technol. A.

[67] Hwang D.H., Nomura A., Kim J., Kim J.H., Cho H., Lee C., Ohno K., Tsujii Y. (2012). Synthesis and characterization of polystyrene brushes for organic thin film transistors. J. Nanosci. Nanotechnol..

[68] Oria L., Ruiz De Luzuriaga A., Alduncin J.A., Perez-Murano F. (2013). Polystyrene as a brush layer for directed self-assembly of block co-polymers. Microelectron. Eng..

[69] Durand W.J., Blachut G., Maher M.J., Sirard S., Tein S., Carlson M.C., Asano Y., Zhou S.X., Lane A.P., Bates C.M. (2015). Design of High- v Block Copolymers for Lithography. J. Polym. Sci. Part A Polym. Chem..

[70] Nakajima H., Dijkstra P., Loos K. (2017). The recent developments in biobased polymers toward general and engineering applications: Polymers that are upgraded from biodegradable polymers, analogous to petroleum-derived polymers, and newly developed. Polymers (Basel).

[71] Scholten P.B.V., Moatsou D., Detrembleur C., Meier M.A.R. (2020). Progress Toward Sustainable Reversible Deactivation Radical Polymerization. Macromol. Rapid Commun..

[72] Gbabode G., Delvaux M., Schweicher G., Andreasen J.W., Nielsen M.M., Geerts Y.H. (2017). Unique Crystal Orientation of Poly(ethylene oxide) Thin Films by Crystallization Using a Thermal Gradient. Macromolecules.

[73] Miyazaki T., Igarashi K., Matsumoto Y., Cabral H. (2019). One-Pot Synthesis of PEG-Poly(amino acid) Block Copolymers Assembling Polymeric Micelles with PEG-Detachable Functionality. ACS Biomater. Sci. Eng..

[74] Rabotyagova O.S., Cebe P., Kaplan D.L. (2011). Protein-based block copolymers. Biomacromolecules.

[75] Banta R.A., Collins T.W., Curley R.A., Young P.W., Holmes J.D., Flynn E.J. (2018). Nanopatterned protein-polysaccharide thin films by humidity regulated phase separation. J. Colloid Interface Sci..

[76] Jeon C.W., Park S., Bang J.H., Chae S., Song K., Lee S.W. (2018). Nonpolar surface modification using fatty acids and its effect on calcite from mineral carbonation of desulfurized gypsum. Coatings.

[77] Micciulla S., Hayward D.W., Gerelli Y., Panzarella A., von Klitzing R., Gradzielski M., Chiappisi L. (2019). One-step procedure for the preparation of functional polysaccharide/fatty acid multilayered coatings. Commun. Chem..

[78] Wang S., Vajjala Kesava S., Gomez E.D., Robertson M.L. (2013). Sustainable thermoplastic elastomers derived from fatty acids. Macromolecules.

[79] Buzzacchera I., Vorobii M., Kostina N.Y., De Los Santos Pereira A., Riedel T., Bruns M., Ogieglo W., Möller M., Wilson C.J., Rodriguez-Emmenegger C. (2017). Polymer Brush-Functionalized Chitosan Hydrogels as Antifouling Implant Coatings. Biomacromolecules.

[80] Mello R.S., Bedendo G.C., Nome F., Fiedler H.D., Laranjeira M.C.M. (2006). Preparation of chitosan membranes for filtration and concentration of compounds under high pressure process. Polym. Bull..

[81] Swain S.N., Biswal S.M., Nanda P.K., Nayak P.L. (2004). Biodegradable Soy-Based Plastics: Opportunities and Challenges. J. Polym. Environ..

[82] Sessa D.J., Mohamed A., Byars J.A., Hamaker S.A.H., Selling G.W. (2007). Properties of films from corn zein reacted with glutaraldehyde. J. Appl. Polym. Sci..

[83] Horniblow R.D., Dowle M., Iqbal T.H., Latunde-Dada G.O., Palmer R.E., Pikramenou Z., Tselepis C. (2015). Alginate-iron speciation and its effect on in vitro cellular iron metabolism. PLoS ONE.

[84] Braun H.G., Meyer E. (2013). Structure formation of ultrathin PEO films at solid interfaces-complex pattern formation by dewetting and crystallization. Int. J. Mol. Sci..

[85] Wang S., Chen F., Song X. (2015). Preparation and characterization of lignin-based membrane material. BioResources.

[86] Shrotri A., Kobayashi H., Fukuoka A. (2017). Catalytic Conversion of Structural Carbohydrates and Lignin to Chemicals. Advances in Catalysis.

[87] Hong M., Chen E.Y.X. (2016). Completely recyclable biopolymers with linear and cyclic topologies via ring-opening polymerization of γ-butyrolactone. Nat. Chem..

[88] Nazarzadeh Zare E., Mansour Lakouraj M., Mohseni M. (2014). Biodegradable polypyrrole/dextrin conductive nanocomposite: Synthesis, characterization, antioxidant and antibacterial activity. Synth. Met..

[89] Sun C., Gao L., Wang D., Zhang M., Liu Y., Geng Z., Xu W., Liu F., Bian H. (2016). Biocompatible polypyrrole-block copolymer-gold nanoparticles platform for determination of inosine monophosphate with bi-enzyme biosensor. Sens. Actuators B Chem..

[90] Mondal D., Mollick M.M.R., Bhowmick B., Maity D., Bain M.K., Rana D., Mukhopadhyay A., Dana K., Chattopadhyay D. (2013). Effect of poly(vinyl pyrrolidone) on the morphology and physical properties of poly(vinyl alcohol)/sodium montmorillonite nanocomposite films. Prog. Nat. Sci. Mater. Int..

[91] Kumar N., Ravikumar M.N.V., Domb A.J. (2001). Biodegradable block copolymers. Adv. Drug Deliv. Rev..

[92] Kreiger M., Pearce J.M. (2013). Environmental life cycle analysis of distributed three-dimensional printing and conventional manufacturing of polymer products. ACS Sustain. Chem. Eng..

[93] Morão A., de Bie F. (2019). Life Cycle Impact Assessment of Polylactic Acid (PLA) Produced from Sugarcane in Thailand. J. Polym. Environ..

[94] Gironi V.P.S.S.F. (2013). Chemical Recycling of PLA: A Great Opportunity Towards the Sustainable Development?. J. Polym. Environ..

[95] Erdmenger T., Guerrero-Sanchez C., Vitz J., Hoogenboom R., Schubert U.S. (2010). Recent developments in the utilization of green solvents in polymer chemistry. Chem. Soc. Rev..

[96] Renoud P., Toury B., Benayoun S., Attik G., Grosgogeat B. (2012). Functionalization of titanium with chitosan via silanation: Evaluation of biological and mechanical performances. PLoS ONE.

[97] Mayeda M.K., Hayat J., Epps T.H., Lauterbach J. (2015). Metal oxide arrays from block copolymer thin film templates. J. Mater. Chem. A.

[98] Lundy R., Flynn S.P., Cummins C., Kelleher S.M., Collins M.N., Dalton E., Daniels S., Morris M.A., Enright R. (2017). Controlled solvent vapor annealing of a high: χ block copolymer thin film. Phys. Chem. Chem. Phys..

[99] Asatekin A., Barr M.C., Baxamusa S.H., Lau K.K.S., Tenhaeff W., Xu J., Gleason K.K. (2010). Designing polymer surfaces via vapor deposition. Mater. Today.

[100] Moni P., Al-Obeidi A., Gleason K.K. (2017). Vapor deposition routes to conformal polymer thin films. Beilstein J. Nanotechnol..

[101] Pierson H.O. (1999). Handbook of Chemical Vapor Deposition (CVD) Principles, Technology, and Applications.

[102] Choy K. (2003). Chemical vapour deposition of coatings. Prog. Mater. Sci..

[103] Gleason K.K. (2020). Nanoscale control by chemically vapour-deposited polymers. Nat. Rev. Phys..

[104] Reeja-Jayan B., Kovacik P., Yang R., Sojoudi H., Ugur A., Kim D.H., Petruczok C.D., Wang X., Liu A., Gleason K.K. (2014). A Route Towards Sustainability Through Engineered Polymeric Interfaces. Adv. Mater. Interfaces.

[105] Qiu M., Chen X., Fan Y., Xing W. (2017). 1.11 Ceramic Membranes. Compr. Membr. Sci. Eng..

[106] Peng S., Bhushan B. (2012). Smart polymer brushes and their emerging applications. RSC Adv..

[107] Ghoshal T., O’Connell J., Sinturel C., Andreazza P., Holmes J.D., Morris M.A. (2019). Solvent mediated inclusion of metal oxide into block copolymer nanopatterns: Mechanism of oxide formation under UV-Ozone treatment. Polymer (Guildf).

[108] Brassat K., Lindner J.K.N. (2020). Nanoscale Block Copolymer Self-Assembly and Microscale Polymer Film Dewetting: Progress in Understanding the Role of Interfacial Energies in the Formation of Hierarchical Nanostructures. Adv. Mater. Interfaces.

[109] Gu X. (2014). Self-assembly of Block copolymers by solvent vapor annealing, mechanism and lithographic applications. Ph.D. Thesis.

[110] Sinturel C., Vayer M., Morris M., Hillmyer M.A. (2013). Solvent vapor annealing of block polymer thin films. Macromolecules.

[111] Gu X., Gunkel I., Hexemer A., Russell T.P. (2016). Controlling Domain Spacing and Grain Size in Cylindrical Block Copolymer Thin Films by Means of Thermal and Solvent Vapor Annealing. Macromolecules.

[112] Höfer R., Bigorra J. (2007). Green chemistry—a sustainable solution for industrial specialties applications. Green Chem..

[113] Chavis M.A., Smilgies D.M., Wiesner U.B., Ober C.K. (2015). Widely tunable morphologies in block copolymer thin films through solvent vapor annealing using mixtures of selective solvents. Adv. Funct. Mater..

[114] Gotrik K.W., Ross C.A. (2013). Solvothermal annealing of block copolymer thin films. Nano Lett..

[115] Gotrik K.W., Hannon A.F., Son J.G., Keller B., Alexander-Katz A., Ross C.A. (2012). Morphology control in block copolymer films using mixed solvent vapors. ACS Nano.

[116] Cummins C., Kelly R.A., Gangnaik A., Georgiev Y.M., Petkov N., Holmes J.D., Morris M.A. (2015). Solvent vapor annealing of block copolymers in confined topographies: Commensurability considerations for nanolithography. Macromol. Rapid Commun..

[117] Nikles S.M., Piao M., Lane A.M., Nikles D.E. (2001). Ethyl lactate: A green solvent for magnetic tape coating. Green Chem..

[118] Kua Y.L., Gan S., Ng H.K., Morris A. The potential of ethyl lactate as a green solvent to extract carotenoids and vitamin e from crude palm oil. Proceedings of the ISPFVF.

[119] Jessop P.G. (2011). Searching for green solvents. Green Chem..

[120] Byrne F.P., Jin S., Paggiola G., Petchey T.H.M., Clark J.H., Farmer T.J., Hunt A.J., Robert McElroy C., Sherwood J. (2016). Tools and techniques for solvent selection: Green solvent selection guides. Sustain. Chem. Process..

[121] Amelio A., Genduso G., Vreysen S., Luis P., Van Der Bruggen B. (2014). Guidelines based on life cycle assessment for solvent selection during the process design and evaluation of treatment alternatives. Green Chem..

[122] Mallakpour S., Rafiee Z. (2011). Ionic Liquids as Environmentally Friendly Solvents in Macromolecules Chemistry and Technology, Part I. J. Polym. Environ..

[123] Hulkkonen H., Salminen T., Niemi T. (2019). Automated solvent vapor annealing with nanometer scale control of film swelling for block copolymer thin films. Soft Matter.

[124] Cheng X., Böker A., Tsarkova L. (2019). Temperature-controlled solvent vapor annealing of thin block copolymer films. Polymers (Basel).

[125] Selkirk A., Prochukhan N., Lundy R., Cummins C., Gatensby R., Kilbride R., Parnell A., Vasquez J.B., Morris M., Mokarian-tabari P. (2021). Optimization and Control of Large Block Copolymer Self-Assembly via Precision Solvent Vapor Annealing. Macromolecules.

[126] Wan Z., Lee H.J., Kim H.G., Jo G.C., Park W.I., Ryu S.W., Lee H., Kwon S. (2018). Circular Double-Patterning Lithography Using a Block Copolymer Template and Atomic Layer Deposition. Adv. Mater. Interfaces.

[127] Singer J.P., Gotrik K.W., Lee J.H., Kooi S.E., Ross C.A., Thomas E.L. (2014). Alignment and reordering of a block copolymer by solvent-enhanced thermal laser direct write. Polymer (Guildf).

[128] Leniart A.A., Pula P., Sitkiewicz A., Majewski P.W. (2020). Macroscopic Alignment of Block Copolymers on Silicon Substrates by Laser Annealing. ACS Nano.

[129] Johnson R.W., Hultqvist A., Bent S.F. (2014). A brief review of atomic layer deposition: From fundamentals to applications. Mater. Today.

[130] Cummins C., Morris M.A. (2018). Using block copolymers as infiltration sites for development of future nanoelectronic devices: Achievements, barriers, and opportunities. Microelectron. Eng..

[131] Subramanian A., Doerk G., Kisslinger K., Yi D.H., Grubbs R.B., Nam C.-Y. (2019). Three-dimensional electroactive ZnO nanomesh directly derived from hierarchically self-assembled block copolymer thin films. Nanoscale.

[132] Subramanian A., Tiwale N., Doerk G., Kisslinger K., Nam C.Y. (2020). Enhanced Hybridization and Nanopatterning via Heated Liquid-Phase Infiltration into Self-Assembled Block Copolymer Thin Films. ACS Appl. Mater. Interfaces.

[133] Snelgrove M., McFeely C., Mani-Gonzalez P.G., Lahtonen K., Lundy R., Hughes G., Valden M., McGlynn E., Yadav P., Saari J. (2020). Aluminium oxide formation via atomic layer deposition using a polymer brush mediated selective infiltration approach. Appl. Surf. Sci..

[134] Ghoshal T., Shaw M.T., Holmes J.D., Morris M.A. (2016). Development of a facile block copolymer method for creating hard mask patterns integrated into semiconductor manufacturing. Nano Res..

[135] Park J.T., Koh J.H., Lee K.J., Seo J.A., Min B.R., Kim J.H. (2008). Formation of silver nanoparticles created in situ in an amphiphilic block copolymer film. J. Appl. Polym. Sci..

[136] Akinoglu G.E., Mir S.H., Gatensby R., Rydzek G., Mokarian-Tabari P. (2020). Block Copolymer Derived Vertically Coupled Plasmonic Arrays for Surface-Enhanced Raman Spectroscopy. ACS Appl. Mater. Interfaces.

[137] Pease R.F., Chou S.Y. (2008). Lithography and Other Patterning Techniques for Future Electronics. Proc. IEEE.

[138] (2018). IEEE International Roadmap for Devices and Systems 2017 - Emerging Research Matterials.

[139] Liddle J.A., Bowser J., Ilic B.R., Luciani V. (2020). So, You Want to Have a Nanofab? Shared-Use Nanofabrication and Characterization Facilities: Cost-of-Ownership, Toolset, Utilization, and Lessons Learned. J. Res. Natl. Inst. Stand. Technol..

[140] Wan L., Ruiz R., Gao H., Patel K.C., Albrecht T.R., Yin J., Kim J., Cao Y., Lin G. (2015). The Limits of Lamellae-Forming PS- b -PMMA Block Copolymers for Lithography. ACS Nano.

[141] Sreenivasan S.V. (2017). Nanoimprint lithography steppers for volume fabrication of leading-edge semiconductor integrated circuits. Microsyst. Nanoeng..

[142] Higashiki T. (2011). Nanoimprint lithography and future patterning for semiconductor devices. J. Micro/Nanolithography MEMS MOEMS.

[143] (2020). AXA XL Risk Consulting Semiconductor Manufacturing.

[144] Naulleau P. (2019). Optical Lithography. Comprehensive Nanoscience and Nanotechnology.

[145] Sanders D.P. (2010). Advances in Patterning Materials for 193 nm Immersion Lithography. Chem. Rev..

[146] Vigneswaran N., Samsuri F., Ranganathan B. (2014). Padmapriya Recent Advances in Nano Patterning and Nano Imprint Lithography for Biological Applications. Procedia Eng..

[147] (2018). Manipulation and Patterning of Surfaces (Nanolithography). Fundamentals and Applications of Nano Silicon in Plasmonics and Fullerines.

[148] Mack C.A. (2006). Field Guide to Optical Lithography.

[149] Huang S.Z., Wu K.Y. (2019). Health Risk Assessment of Photoresists Used in an Optoelectronic Semiconductor Factory. Risk Anal..

[150] Museau M., Tichkiewitch S. (2007). Integrated design of mems: Aiming at manufacturability. Int. J. Interact. Des. Manuf. (IJIDeM).

[151] Chaniago Y.D., Hussain A., Andika R., Lee M. (2019). Reactive Pressure-Swing Distillation toward Sustainable Process of Novel Continuous Ultra-High-Purity Electronic-Grade Propylene Glycol Monomethyl Ether Acetate Manufacture. ACS Sustain. Chem. Eng..

[152] Wang Y., Meng F., Lin Y., Duan W., Liu Q. (2017). Chemosphere Four types of attenuation of phenol and cresols in microcosms under simulated marine conditions: A kinetic study. Chemosphere.

[153] Bahadar H., Mostafalou S., Abdollahi M. (2014). Current understandings and perspectives on non-cancer health effects of benzene: A global concern. Toxicol. Appl. Pharmacol..

[154] van de Kerkhof M.A., Benschop J.P.H., Banine V.Y. (2019). Lithography for now and the future. Solid. State. Electron..

[155] Wong A.K. (2003). Microlithography: Trends, challenges, solutions, and their impact on design. IEEE Micro.

[156] Chen Y., Xiong S. (2020). Directed self-assembly of block copolymers for sub-10 nm fabrication. Int. J. Extrem. Manuf..

[157] Wang C.H., Huang C.Y., Yak H.K., Hsieh H.C., Wang J.L. (2021). Identifying an unknown compound in flue gas of semiconductor industry – Forensics of a perfluorocarbon. Chemosphere.

[158] Chang M.B., Chang J.S., Chang M.B., Chang J.S. (2006). Abatement of PFCs from Semiconductor Manufacturing Processes by Nonthermal Plasma Technologies: A Critical Review. Ind. Eng. Chem. Res..

[159] Mariussen E., Fonnum F., Mariussen E. (2008). Critical Reviews in Toxicology Neurochemical Targets and Behavioral Effects of Organohalogen Compounds: An Update Neurochemical Targets and Behavioral Effects of Organohalogen Compounds: An Update. Crit. Rev. Toxicol..

[160] Dietz R., Gustavson K., Sonne C., Desforges J., Rigét F.F., Pavlova V., Mckinney M.A., Letcher R.J. (2015). Physiologically-based pharmacokinetic modelling of immune, re- productive and carcinogenic effects from contaminant exposure in polar bears (Ursus maritimus) across the Arctic. Environ. Res..

[161] Murata T., Ohtsuka M., Hotta K., Fujisawa M. Conversion from helium to nitrogen as a TEOS carrier gas in sub-atmospheric chemical vapor deposition. Proceedings of the 2013 e-Manufacturing & Design Collaboration Symposium (eMDC).

[162] Wu B., Kumar A. (2007). Extreme ultraviolet lithography: A review. J. Vac. Sci. Technol. B Microelectron. Nanom. Struct..

[163] Ghosh S., Satyanarayana V.S.V., Pramanick B., Sharma S.K., Pradeep C.P., Morales-Reyes I., Batina N., Gonsalves K.E. (2016). Patterning highly ordered arrays of complex nanofeatures through EUV directed polarity switching of non chemically amplified photoresist. Sci. Rep..

[164] van de Kerkhof M., Jasper H., Levasier L., Peeters R., van Es R., Bosker J.-W., Zdravkov A., Lenderink E., Evangelista F., Broman P., Panning E.M., Goldberg K.A. (2017). Enabling sub-10nm node lithography: Presenting the NXE:3400B EUV scanner. Proceedings of the SPIE.

[165] Choi S., Park D., Park Y. (2021). Possibility of Benzene Exposure in Workers of a Semiconductor Industry Based on the Patent Resources, 1990–2010. Saf. Health Work.

[166] Turkot B., Carson S., Lio A. (2018). Continuing moore’s law with EUV lithography. Tech. Dig. Int. Electron Devices Meet. IEDM.

[167] Van Schoot J., Schift H. (2017). Next-generation lithography–an outlook on EUV projection and nanoimprint. Adv. Opt. Technol..

[168] Itani T., Kozawa T. (2013). Resist Materials and Processes for Extreme Ultraviolet Lithography. Jpn. J. Appl. Phys..

[169] De Silva A., Felix N.M., Ober C.K. (2008). Molecular Glass Resists as High-Resolution Patterning Materials. Adv. Mater..

[170] Mojarad N., Gobrecht J., Ekinci Y. (2015). Beyond EUV lithography: A comparative study of efficient photoresists’ performance. Sci. Rep..

[171] Barcelo S., Li Z. (2016). Nanoimprint lithography for nanodevice fabrication. Nano Converg..

[172] Havard J.M., Shim S.Y., Fréchet J.M.J., Lin Q., Medeiros D.R., Grant Willson C., Byers J.D. (1999). Design of photoresists with reduced environmental impact. 1. Water-soluble resists based on photo-cross-linking of poly(vinyl alcohol). Chem. Mater..

[173] Tormen M., Sovernigo E., Pozzato A., Pianigiani M., Tormen M. (2015). Sub-100 l s nanoimprint lithography at wafer scale. Microelectron. Eng..

[174] Cox L.M., Martinez A.M., Blevins A.K., Sowan N., Ding Y., Bowman C.N. (2020). Nanoimprint lithography: Emergent materials and methods of actuation. Nano Today.

[175] Kwon B., Kim J.H. (2016). Importance of Molds for Nanoimprint Lithography: Hard, Soft, and Hybrid Molds. J. Nanosci..

[176] Schift H. (2008). Nanoimprint lithography: An old story in modern times? A review. J. Vac. Sci. Technol. B Microelectron. Nanom. Struct..

[177] Nicaise S.M., Tavakkoli K.G.A., Berggren K.K. (2015). Self-Assembly of Block Copolymers by Graphoepitaxy.

[178] Doble M., Kruthiventi A.K. (2007). Industrial Examples. Green Chemistry and Engineering.

[179] Iacopi F., McIntosh M. (2019). Opportunities and perspectives for green chemistry in semiconductor technologies. Green Chem..

[180] Shen C., Tran P., Minh Ly P. (2018). Chemical Waste Management in the U.S. Semiconductor Industry. Sustainability.

[181] Schischke K., Stutz M., Ruelle J.-P., Griese H., Reichl H. Life cycle inventory analysis and identification of environmentally significant aspects in semiconductor manufacturing. Proceedings of the 2001 IEEE International Symposium on Electronics and the Environment. 2001 IEEE ISEE (Cat. No.01CH37190).

[182] Hsu L., Huang C., Chuang Y., Chen H., Chan Y. (2016). Accumulation of heavy metals and trace elements in fluvial sediments received effluents from traditional and semiconductor industries. Nat. Publ. Gr..

[183] Suzuki Y., Watanabe I., Oshida T., Chen Y. (2007). Accumulation of trace elements used in semiconductor industry in Formosan squirrel, as a bio-indicator of their exposure, living in Taiwan. Chemosphere.

[184] Chaniago Y.D., Minh L.Q., Khan M.S., Koo K.-K., Bahadori A., Lee M. (2015). Optimal design of advanced distillation configuration for enhanced energy efficiency of waste solvent recovery process in semiconductor industry. Energy Convers. Manag..

[185] Sun C., Rose T. (2015). Supply Chain Complexity in the Semiconductor Industry: Assessment from System View and the Impact of Changes. IFAC-PapersOnLine.

[186] Hu S.-C., Tsai Y.-W., Fu B.-R., Chang C.-K. (2017). Assessment of the SEMI energy conversion factor and its application for semiconductor and LCD fabs. Appl. Therm. Eng..

[187] Hu S.-C., Lin T., Huang S.-H., Fu B.-R., Hu M.-H. (2020). Energy savings approaches for high-tech manufacturing factories. Case Stud. Therm. Eng..

[188] (2003). ITRS International Technology Roadmap for Semiconductors, 2003 Edition Enviornment, saftey and health.

[189] Denkena B., Abele E., Brecher C., Dittrich M.-A., Kara S., Mori M. (2020). Energy efficient machine tools. CIRP Ann..

[190] Wang C.T., Chiu C.S. (2014). Competitive strategies for Taiwan’s semiconductor industry in a new world economy. Technol. Soc..

[191] Zimmerman J.B., Anastas P.T., Erythropel H.C., Leitner W. (2020). Designing for a green chemistry future. Science.

[192] Lin A.Y., Panchangam S.C., Lo C. (2009). The impact of semiconductor, electronics and optoelectronic industries on downstream perfluorinated chemical contamination in Taiwanese rivers. Environ. Pollut..

[193] Loganathan B.G. (2017). Author’s personal copy. Int. Encycl. Public Health.

[194] Liu S., Xia T. (2020). Continued Efforts on Nanomaterial-Environmental Health and Safety Is Critical to Maintain Sustainable Growth of Nanoindustry. Small.

[195] Adam V., Nowack B. (2017). European country-specific probabilistic assessment of nanomaterial flows towards landfilling, incineration and recycling. Environ. Sci. Nano.

[196] Watjanatepin P., Castagnola V., Cetin Y., Linkov I., Skentelbery C., Prodanov D. (2020). Workshop Report: Governance of Emerging Nanotechnology Risks in the Semiconductor Industry. Front. Public Health.

[197] Miseljic M., Olsen S.I. (2014). Life-cycle assessment of engineered nanomaterials: A literature review of assessment status. J. Nanoparticle Res..

[198] Malakar A., Kanel S.R., Ray C., Snow D.D., Nadagouda M.N. (2020). Nanomaterials in the environment, human exposure pathway, and health effects: A review. Sci. Total Environ..

[199] Johnston L.J., Gonzalez-Rojano N., Wilkinson K.J., Xing B. (2020). Key challenges for evaluation of the safety of engineered nanomaterials. NanoImpact.

[200] Kim S., Yoon C., Ham S., Park J., Kwon O., Park D., Choi S., Kim S., Ha K., Kim W. (2018). Chemical use in the semiconductor manufacturing industry. Int. J. Occup. Environ. Health.

[201] Espinosa N., Hösel M., Jørgensen M., Krebs F.C. (2014). Large scale deployment of polymer solar cells on land, on sea and in the air. Energy Environ. Sci..

[202] (2020). IRDS International Roadmap For Devices and Systems 2020 Edition Factory Integration.

[203] Anastas P.T., Kirchhoff M.M. (2002). Origins, current status, and future challenges of green chemistry. Acc. Chem. Res..

[204] Tufvesson L.M., Tufvesson P., Woodley J.M., Börjesson P. (2013). Life cycle assessment in green chemistry: Overview of key parameters and methodological concerns. Int. J. Life Cycle Assess..

[205] (2010). European Commission -- Joint Research Centre -- Institute for Environment and Sustainability International Reference Life Cycle Data System (ILCD) Handbook -- General guide for Life Cycle Assessment -- Detailed guidance.

[206] Choi K.-H., Kim H., Kim M.-H., Kwon H.-J. (2019). Semiconductor Work and Adverse Pregnancy Outcomes Associated with Male Workers: A Retrospective Cohort Study. Ann. work Expo. Health.

[207] Plepys A. (2004). The environmental impacts of electronics. Going beyond the walls of semiconductor fabs. IEEE Int. Symp. Electron. Environ..

[208] Steffen W., Rockström J., Richardson K., Lenton T.M., Folke C., Liverman D. (2018). Trajectories of the Earth System in the Anthropocene. Proc. Natl. Acad. Sci. USA.

[209] Kim J.H., Jin H.M., Yang G.G., Han K.H., Yun T., Shin J.Y., Jeong S., Kim S.O. (2020). Smart Nanostructured Materials based on Self-Assembly of Block Copolymers. Adv. Funct. Mater..

[210] Su J.C.P., Wang L., Ho J.C. (2016). The timing of green product introduction in relation to technological evolution. J. Ind. Prod. Eng..

[211] Ambec S., Lanoie P. (2008). Does It Pay to Be Green? A Systematic Overview. Acad. Manag. Perspect..

[212] Kim H., Kwon H.J., Rhie J., Lim S., Kang Y.D., Eom S.Y., Lim H., Myong J.P., Roh S. (2017). The relationship between spontaneous abortion and female workers in the semiconductor industry. Ann. Occup. Environ. Med..

[213] Correa A., Gray R.H., Cohen R., Rothman N., Shah F., Seacat H., Corn M. (1996). Ethylene glycol ethers and risks of spontaneous abortion and subfertility. Am. J. Epidemiol..

[214] Fomenkov I., Brandt D., Ershov A., Schafgans A., Tao Y., Vaschenko G., Rokitski S., Kats M., Vargas M., Purvis M. (2017). Light sources for high-volume manufacturing EUV lithography: Technology, performance, and power scaling. Adv. Opt. Technol..

[215] Tsai W.-T. (2011). Environmental and health risks of chlorine trifluoride (ClF3), an alternative to potent greenhouse gases in the semiconductor industry. J. Hazard. Mater..

[216] Jin C., Olsen B.C., Luber E.J., Buriak J.M. (2017). Nanopatterning via Solvent Vapor Annealing of Block Copolymer Thin Films. Chem. Mater..

[217] Eng C.Y., Yan D., Withanage N., Liang Q., Zhou Y. (2019). Wastewater treatment and recycle from a semiconductor industry: A demo-plant study. Water Pract. Technol..

[218] Chung J., Fleege D., Ong S.K., Lee Y. (2015). Organic semiconductor wastewater treatment using a four-stage Bardenpho with membrane system. Environ. Technol..

[219] Tang C.Y., Fu Q.S., Robertson A.P., Criddle C.S., Leckie J.O. (2006). Use of reverse osmosis membranes to remove perfluorooctane sulfonate (PFOS) from semiconductor wastewater. Environ. Sci. Technol..

[220] Wang Y., Zhang Z., Jiang C., Xu T. (2013). Electrodialysis process for the recycling and concentrating of tetramethylammonium hydroxide (TMAH) from photoresist developer wastewater. Ind. Eng. Chem. Res..

[221] Lin C.C., Yang C.C., Ger J., Deng J.F., Hung D.Z. (2010). Tetramethylammonium hydroxide poisoning. Clin. Toxicol..

[222] Chang K.F., Yang S.Y., You H.S., Pan J.R. (2008). Anaerobic treatment of tetra-methyl ammonium hydroxide (TMAH) containing wastewater. IEEE Trans. Semicond. Manuf..

[223] Kim E.A., Lee H.E., Ryu H.W., Park S.H., Kang S.K. (2011). Cases series of malignant lymphohematopoietic disorder in Korean semiconductor industry. Saf. Health Work.

[224] Kim M.-H., Kim H., Paek D. (2014). The health impacts of semiconductor production: An epidemiologic review. Int. J. Occup. Environ. Health.

[225] Thompson L.F. (1983). An Introduction to Lithography.

[226] Puliyalil H., Cvelbar U. (2016). Selective plasma etching of polymeric substrates for advanced applications. Nanomaterials.

[227] Martinez V.M., Edgar T.F. (2006). IEEE Control Systems Magazine.

[228] Santillan J.J., Harumoto M., Motono T., Dos Santos A.F., Mori C., Tanaka Y., Stokes H., Asai M., Itani T. (2021). Application of ethyltrimethylammonium hydroxide (ETMAH) as an alternative developer solution/process for semiconductor lithography. Jpn. J. Appl. Phys..

[229] Mori I.C., Arias-Barreiro C.R., Koutsaftis A., Ogo A., Kawano T., Yoshizuka K., Inayat-Hussain S.H., Aoyama I. (2015). Toxicity of tetramethylammonium hydroxide to aquatic organisms and its synergistic action with potassium iodide. Chemosphere.

[230] Niu X., Field J.A., Paniego R., Pepel R.D., Chorover J., Abrell L., Sierra-alvarez R. (2021). Bioconcentration potential and microbial toxicity of onium cations in photoacid generators PAG-2. Environ. Sci. Pollut. Res..

[231] Ober M.S., Romer D.R., Etienne J., Thomas P.J., Jain V., Cameron J.F., Thackeray J.W. (2019). Backbone Degradable Poly(aryl acetal) Photoresist Polymers: Synthesis, Acid Sensitivity, and Extreme Ultraviolet Lithography Performance. Macromolecules.

[232] Knaepen W., American T., For S., Mallik A., Vandenbroeck N. Improved cost-effectiveness of the block co-polymer anneal process for DSA. Proceedings of the Alternative Lithographic Technologies VIII.

[233] Louria D.B., Joselow M.M., Browder A.A. (1972). The Human Toxicity of Certain Trace Elements. Ann. Intern. Med..

[234] Chung S., Chung J., Chung C. (2020). Journal of Water Process Engineering Enhanced electrochemical oxidation process with hydrogen peroxide pretreatment for removal of high strength ammonia from semiconductor wastewater. J. Water Process Eng..

[235] Inoue K. (2013). Heavy metal toxicity. J. Clin. Toxicol..

[236] Klopffer W., Klöpffer W. (2014). Background and Future Prospects in Life Cycle Assessment.

[237] (2006). International Organization for Standardization ISO 14040: Environmental management—Life cycle assessment—Principles and framework.

[238] Mutel C., Liao X., Patouillard L., Bare J., Fantke P., Frischknecht R., Hauschild M., Jolliet O., Maia de Souza D., Laurent A. (2019). Overview and recommendations for regionalized life cycle impact assessment. Int. J. Life Cycle Assess..

[239] García-Valverde R., Cherni J.A., Urbina A. (2010). Life cycle analysis of organic photovoltaic technologies. Prog. Photovoltaics Res. Appl..

[240] Moni S.M., Mahmud R., High K., Carbajales-Dale M. (2020). Life cycle assessment of emerging technologies: A review. J. Ind. Ecol..

[241] Tecchio P., Freni P., De Benedetti B., Fenouillot F. (2016). Ex-ante Life Cycle Assessment approach developed for a case study on bio-based polybutylene succinate. J. Clean. Prod..

[242] Buyle M., Audenaert A., Billen P., Boonen K., Van Passel S. (2019). The Future of Ex-Ante LCA? Lessons Learned and Practical Recommendations. Sustainability.

[243] Tsoy N., Steubing B., van der Giesen C., Guinée J. (2020). Upscaling methods used in ex ante life cycle assessment of emerging technologies: A review. Int. J. Life Cycle Assess..

[244] van der Giesen C., Cucurachi S., Guinée J., Kramer G.J., Tukker A. (2020). A critical view on the current application of LCA for new technologies and recommendations for improved practice. J. Clean. Prod..

[245] Thonemann N., Schulte A., Maga D. (2020). How to Conduct Prospective Life Cycle Assessment for Emerging Technologies? A Systematic Review and Methodological Guidance. Sustainability.

[246] Kirchain R.E., Gregory J.R., Olivetti E.A. (2017). Environmental life-cycle assessment. Nat. Mater..

[247] Göswein V., Habert G., Rodrigues C., König J., Silvestre J.D., Freire F. (2021). Using anticipatory life cycle assessment to enable future sustainable construction. J. Ind. Ecol..

[248] Piccinno F., Hischier R., Seeger S., Som C. (2016). From laboratory to industrial scale: A scale-up framework for chemical processes in life cycle assessment studies. J. Clean. Prod..

[249] Zhang X., Zhang L., Yuan Y., Zhai Q. (2020). Life Cycle Assessment on Wave and Tidal Energy Systems: A Review of Current Methodological Practice. Int. J. Environ. Res. Public Health.

[250] (2006). International Organization for Standardization ISO 14044: Environmental Management: Life Cycle Assessment; Principles and Framework.

[251] Finnveden G., Hauschild M.Z., Ekvall T., Guinée J., Heijungs R., Hellweg S., Koehler A., Pennington D., Suh S. (2009). Recent developments in Life Cycle Assessment. J. Environ. Manag..

[252] Hauschild M.Z., Rosenbaum R.K., Olsen S.I., Hauschild M.Z., Rosenbaum R.K., Olsen S.I. (2018). LCA Cookbook. Life Cycle Assessment.

[253] Ramanathan M., Tseng Y.C., Ariga K., Darling S.B. (2013). Emerging trends in metal-containing block copolymers: Synthesis, self-assembly, and nanomanufacturing applications. J. Mater. Chem. C.

[254] Clift R., Sim S., King H., Chenoweth J., Christie I., Clavreul J., Mueller C., Posthuma L., Boulay A.-M., Chaplin-Kramer R. (2017). The Challenges of Applying Planetary Boundaries as a Basis for Strategic Decision-Making in Companies with Global Supply Chains. Sustainability.

[255] Hischier R., Salieri B., Pini M. (2017). NanoImpact Most important factors of variability and uncertainty in an LCA study of nanomaterials–Findings from a case study with nano titanium dioxide. NanoImpact.

[256] Cooper J.S. (2002). LeA Me! hodology with Case Studies Specifying Functional Units and Reference Flows for Comparable Alternatives. Int. J. Life Cycle Assess..

[257] Bull J.G., Kozak R.A. (2014). Comparative life cycle assessments: The case of paper and digital media. Environ. Impact Assess. Rev..

[258] Korea S. (2006). Critical Review System Boundary Selection in Life-Cycle Inventories Using Hybrid Approaches. Environ. Sci. Technol..

[259] Suh S., Huppes G. (2009). Methods in the Life Cycle Inventory of a Product. Handbook of Input-Output Economics in Industrial Ecology.

[260] Espinosa N., García-Valverde R., Krebs F.C. (2011). Life-cycle analysis of product integrated polymer solar cells. Energy Environ. Sci..

[261] Das S., Mao E. (2020). The global energy footprint of information and communication technology electronics in connected Internet-of-Things devices. Sustain. Energy Grids Netw..

[262] Aymard V., Botta-Genoulaz V. (2017). Normalisation in life-cycle assessment: Consequences of new European factors on decision-making. Supply Chain Forum.

[263] Lesage P., Muller S. (2017). Life Cycle Inventory: An In-Depth Look at the Modeling, Data, and Available Tools.

